# Drug stock-outs in HIV, tuberculosis, and malaria programs: a systematic review

**DOI:** 10.1016/j.eclinm.2026.104119

**Published:** 2026-07-31

**Authors:** Avaneesh Kumar Pandey, Ritika Kondel Bhandari, Esmita Charani, Jennifer Cohn, Amrita Ghautare, Anne-Grete Märtson, Uttara Gadde, Sipho Dlamini, Malcolm Miller, Samir Malhotra, Thomas Tängdén, Sanjeev Singh, Marc Mendelson, Nusrat Shafiq

**Affiliations:** a Clinical Pharmacology Unit, Department of Pharmacology, Post Graduate Institute of Medical Education and Research, Chandigarh, India; b Division of Infectious Diseases & HIV Medicine, Department of Medicine, Groote Schuur Hospital, University of Cape Town, Cape Town, South Africa; c Faculty of Health and Medical Sciences, University of Liverpool, Liverpool, UK; d The Global Antibiotic Research and Development Partnership, Geneva, Switzerland; e Health Protection Research Unit in Healthcare Associated Infections and Antimicrobial Resistance, Department of Medicine, Imperial College London, UK; f Leiden Academic Centre for Drug Research (LACDR) Leiden University, Netherlands; g University of Pennsylvania, USA; h Department of Medical Sciences, Uppsala University, Uppsala, Sweden; i Department of Medical Administration, Amrita Institute of Medical Sciences, Amrita Vishwa Vidyapeetham, Faridabad, Haryana, India

**Keywords:** Anti-infective therapy, Global health, Drug stock-outs, Human immunodeficiency virus (HIV), Tuberculosis (TB), Antimalarials

## Abstract

**Background:**

Stock-outs of antiretroviral, anti-tuberculosis, and anti-malarial medicines remain a major challenge in low- and middle-income countries, affecting both health systems and patient outcomes. This systematic review aimed to synthesize evidence on the causes, consequences, and mitigation strategies related to these stock-outs.

**Methods:**

A systematic search of PubMed, Embase, Web of Science, and Scopus was conducted for studies published between 01, January 2000 and 17, March 2026. Studies reporting stock-outs of antiretroviral or/and anti-tuberculosis or/and anti-malarial medicines were included, while those published before 2000 or focusing on other medicines were excluded. Findings were synthesized using a non-quantitative approach and heterogeneity was assessed qualitatively. The quality of the studies was evaluated using Joanna Briggs Institute (JBI) critical appraisal check list. The review was registered in PROSPERO (CRD42021296472).

**Findings:**

Of 10,158 screened records, 56 studies were included. Key causes of stock-outs included supply chain inefficiencies, procurement delays, inadequate funding, weak governance, and poor demand forecasting. Consequences included treatment interruptions, increased drug resistance, and higher morbidity and mortality, along with increased out-of-pocket costs and emergency procurement burdens. Mitigation strategies ranged from short-term responses, such as emergency purchasing and redistribution, to long-term system strengthening, including improved procurement systems, buffer stocks, supplier diversification, and digital inventory management.

**Interpretation:**

Review demonstrates that the causes of reported stock-outs in HIV, tuberculosis, and malaria programmes are largely preventable and the solutions lie in fortifying supply chain system with better forecasting robust logistics and sufficient financing.

**Funding:**

Wellcome trust CAMO-Net programme and Global Antibiotic Research and Development Partnership.

**Research in context**

**Evidence before this study**

We searched PubMed, Embase, Web of Science and Scopus for studies on medicine stock-outs in Human immunodeficiency virus (HIV), tuberculosis, and malaria programmes in low- and middle-income countries (LMICs), using terms related to stock-outs, supply chains, procurement, and access to medicines since 2000 to March 17, 2026. Existing literature primarily focuses on individual diseases or specific country contexts, with limited cross-disease synthesis. While previous studies have identified supply chain inefficiencies, procurement delays, and financing constraints as key drivers, the evidence remains fragmented and lacks a comprehensive assessment of shared causes, impacts, and mitigation strategies across these major infectious disease programmes.

**Added value of this study**

This systematic review provides a comprehensive synthesis of evidence across HIV, tuberculosis, and malaria programmes, integrating findings from diverse settings and study designs. It identifies common, system-level drivers of stock-outs, including weaknesses in supply chains, procurement processes, and financing structures. The review also highlights the clinical, economic, and psychosocial consequences of stock-outs, and documents both short-term coping mechanisms and longer-term system-level strategies. By examining patterns across diseases and regions, the study provides insights into persistent gaps in forecasting, data systems, and coordinated supply chain management.

**Implications of all the available evidence**

Medicine stock-outs in HIV, tuberculosis, and malaria programmes are primarily driven by preventable health system failures rather than disease-specific factors. Investing in resilient supply chains will improve treatment continuity, reduce the risk of drug resistance, and accelerate progress towards global HIV, tuberculosis, and malaria targets, requiring sustained commitment from policymakers, donors, and health programme managers.

## Introduction

Human immunodeficiency virus (HIV), tuberculosis, and malaria remain leading causes of morbidity and mortality in low- and middle-income countries (LMICs).^1^ Sub-Saharan Africa bears the highest burden, with over 95% of global malaria deaths, and two-thirds of global HIV infections.^1^ Recent uncertainties in global health financing, including reduced or delayed disbursements from key donors such as United States President’s Emergency Plan for AIDS Relief (PEPFAR), The Global Fund, and Gavi, threaten the sustainability of decades of progress. These disruptions increase the risk of stockouts, especially in countries heavily reliant on external aid, and call for stronger national supply chain resilience, diversified financing mechanisms, and real-time data systems.^2^^,^^3^

HIV, tuberculosis, and malaria control programmes depend on uninterrupted access to quality assured drugs. Many LMICs, however, continue to experience periodic medicine shortages and stock-outs, undermining treatment outcomes.^4^, ^5^, ^6^ A stock-out is generally defined as a complete absence of required medicine in stock, resulting in interrupted therapy for patients.^7^ Such supply disruptions are alarmingly common, for example, 67% of surveyed Latin American countries reported at least one antiretroviral stock-out episode in 2010–2011.^7^ Similarly, data from a 15-month national monitoring exercise in Tanzania (2011–2012), an average of 29% of health facilities were completely stocked out of all first-line artemisinin-based combination therapies for malaria (median stock-out duration 6 weeks).^8^

These gaps in medicine supply have serious clinical consequences. Stock-outs of HIV and tuberculosis medicines are known to increase the risk of treatment interruption, drug resistance, treatment failure, and disease progression.^9^^,^^10^ A cohort study in Côte d’Ivoire found that patients who experienced antiretroviral treatment discontinuation due to stock-outs had more than double the risk of death or loss to follow-up compared to those who did not experience stock-outs.^11^ The lack of first-line antimalarials can lead to untreated infections or use of less effective alternatives, potentially resulting in higher malaria morbidity and mortality.^12^

Despite growing recognition of the scale and consequences of medicine stock-outs in LMICs, the available evidence remains fragmented and largely disease-specific. Furthermore, the causes, patterns, and consequences of stock-outs may differ across HIV, tuberculosis, and malaria programmes due to factors such as donor dependence, regimen standardisation, and procurement mechanisms. However, there is a lack of integrated evidence that examines these issues across all three diseases. This systematic review maps the current evidence on stock-outs of HIV, tuberculosis, and malaria medicines, the effects on health systems and patients, and the different strategies that have been suggested or used to prevent them. By bringing together evidence from across all three high disease burden infections, this review aims to identify common challenges, particularly in LMICs in the supply chain and suggested practical ways to ensure that access to these essential medicines is uninterrupted amongst populations that have the highest burden of disease.

## Methods

This systematic review was conducted following Preferred Reporting Items for Systematic Reviews and Meta-Analyses (PRISMA) guidelines identifying studies which have reported on medication shortages or stock-outs in HIV, tuberculosis, and malaria programmes. The systematic review was registered in Prospero [CRD42021296472].

### Search strategy and selection criteria

Three consecutive literature searches were conducted on January 1, 2023, September 1, 2023, and December 1, 2024 across PubMed, Embase, Web of Science, and Scopus to identify studies published from January 1, 2000, up to the respective search dates. Search terms combined synonyms for drug shortages/stock-outs (“stock-out”, “shortage”, “drug supply failure”, “shortfall”, “unavailability”, “withdrawn”) with terms for the diseases of interest (“HIV”, “antiretroviral”, “tuberculosis”, “Tuberculosis”, “malaria”, “antimalarial”) and names of the individual drugs in each category (, “HIV Fusion Inhibitors”, “nucleoside reverse transcriptase inhibitors”, “HIV Protease Inhibitors”, “antitubercular drugs”). The literature search was updated on March 17, 2026, across all databases, with the search period from January 1, 2000, to March 17, 2026. The detailed search strategy is given in Supplementary Table S1. Studies published since January 2000 were eligible for inclusion as this period marks significant changes in global supply chains and procurement systems, including increased reliance on single-supplier models and the concentration of active pharmaceutical ingredient (API) manufacturing in countries such as China and India. There was no language limitation. The review included peer-reviewed articles and relevant reports of any study design (quantitative, qualitative, or mixed-methods) that documented: (1) causes or risk factors for drug shortages/stock-outs, (2) clinical or economic impacts of stock-outs, or (3) strategies adopted or recommended to address stock-outs. Both interventional studies (e.g., evaluations of supply-chain interventions) and observational studies (surveys, cohort studies, etc.) were eligible. Commentaries or editorials unless they presented empirical data on stock-out events and studies published before January 2000. Additional articles were identified by cross referencing of relevant studies and global health reports.

### Study screening and data extraction

Two authors independently screened titles/abstracts for eligibility and reviewed full texts of potentially relevant papers. The methodological quality of included studies was assessed independently by the same two authors using the Joanna Briggs Institute (JBI) Critical Appraisal Tools appropriate to each study design. These design-specific checklists evaluate key domains such as selection bias, measurement validity, confounding, and the appropriateness of data analysis and conclusions. Any discrepancies were resolved by discussing with two additional authors. Studies assessed as having a high risk of bias were not excluded but were considered with caution during interpretation. From each included study, key data was extracted using a standardised form including the following indicators: publication details, study setting and design, list of medicines affected, documented causes of stock-outs, any reported clinical outcomes or economic impacts, and any mitigation measures described. We also noted the disease area (HIV, tuberculosis, malaria) for each study. When available, quantitative indicators such as frequency or duration of stock-outs and patient outcomes were recorded.

### Ethics

As this study involved analysis of previously published and publicly available data, ethics committee approval and informed consent were not required.

### Approach to evidence synthesis

The included studies varied considerably in their objectives, methodologies, measures of stock-outs, and reporting of causes, impacts, and mitigation strategies, precluding meaningful quantitative pooling or meta-analysis. Therefore, a structured narrative synthesis approach was used to summarize the findings of the included studies.

### Role of funding source

The funders had no involvement in study design, data collection, data analyses, data interpretation, or the writing of the report.

## Results

### Characteristics of included studies

**Fig. 1:**
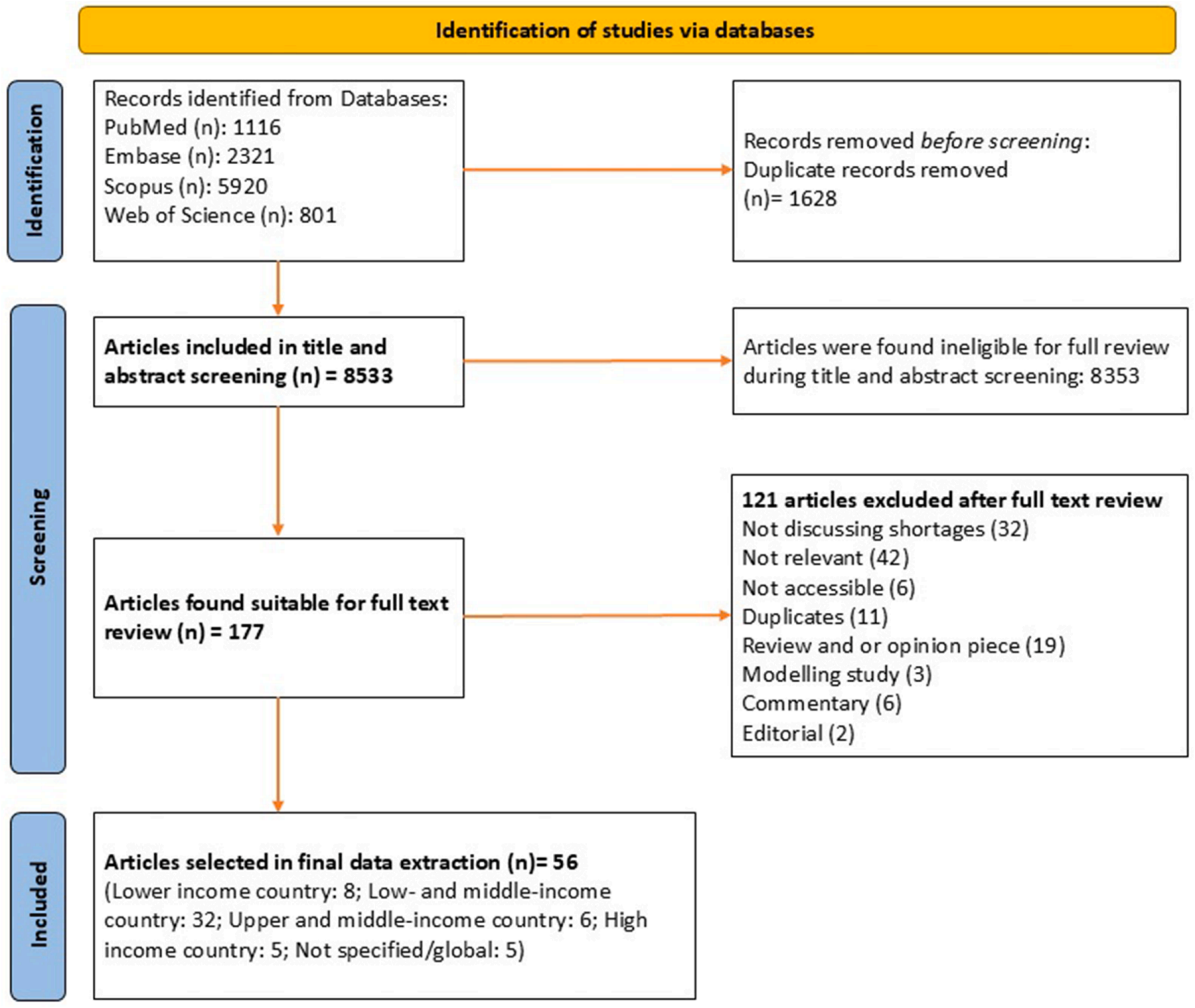
**PRISMA flow diagram of study selection.** Flow diagram illustrating the process of study identification, screening, eligibility assessment, and inclusion. PRISMA: Preferred Reporting Items for Systematic Reviews and Meta-Analyses.

A total of 56 studies met the inclusion criteria, with 27 studies focusing on antiretrovirals, 20 on antimalarials and 9 on anti-tuberculosis medicines. The updated search, performed on March 17, 2026, yielded an additional 4 studies that were eligible for inclusion (Fig. 1; Table 1). The included studies represent diverse geographic regions (Table 2). The majority were from sub-Saharan Africa (n = 40) reflecting the disease burden and number of programmes. There were five studies from Asia and Latin America. The included studies comprised a range of designs, with mixed-methods studies (n = 29) being the most common, followed by qualitative (n = 12), quantitative approaches (n = 10) and other (n = 5) (Table 3). Of the 56 studies included in the review, 55 were assessed for risk of bias using the appropriate JBI Critical Appraisal Checklists based on study design. Among these, 32 studies were judged to have a low risk of bias, 22 were rated as moderate risk, and one study was identified as having a high risk of bias. One study, classified as a news article,^16^ was excluded from quality appraisal due to its non-empirical nature (Supplementary Fig. S1). Considerable heterogeneity was observed across the included studies. The studies varied in design, disease programme, geographical setting, study population, definitions of medicine stock-outs, and outcome reporting. There were also important differences in the reported causes, impacts, and mitigation strategies for stock-outs. Owing to this heterogeneity, the findings were synthesized narratively rather than quantitatively. A review of the reporting patterns of stock-outs in the included studies shows that the majority were published in the last decade (n = 49), compared to only a few in the preceding decade (n = 7).

**Table 1:** Characteristic of included studies in the systematic review of drug stock-outs in human immunodeficiency virus (HIV), tuberculosis, and malaria programs.

Study/year/country	Study design	Population involved	Sample size	List of drugs in shortage
Carmody et al., 2003; Brazil^13^	Retrospective Cross-sectional study with a convenience sample.	Adults living with HIV/AIDS receiving ART	200 participants.	Specific drugs in shortage or stockout were not explicitly identified in the article.
Marcellin et al., 2008; Cameroon^14^	An observational, cross-sectional study conducted as part of the survey.	PLHIV receiving ART	533 individuals	Specific antiretroviral drugs were not explicitly detailed in the sources.
Bernays et al., 2009; Serbia and Montenegro^15^	Qualitative study using in-depth interviews	41 PLHIV- 18 HIV treatment service providers	59 participants in total	Specific drugs were not listed, but the focus was on antiretroviral therapy (ART) necessary for managing HIV.
Henery Wasswa 2009, Uganda^16^	New article in BMJ	HIV patients receiving ARVs	Not specified issue impacts patients nationwide.	ARV medications and Septrin (cotrimoxazole)
Muhamadi et al., 2010; Uganda^17^	A qualitative study combining methods: 1. In-depth interviews and Focus group discussions	PLHIV, healthcare workers, policymakers	Participants for interviews (20) and FGDs (112)	ARVs drugs
Pasquet et al., 2010, Côted’Ivoire^11^	Descriptive observational study	HIV-infected adults on cART	1554 study patients	Nevirapine (NVP), Zidovudine/lamivudine (ZDV-3TC), and other ARVs used in first-line treatment.
Sued et al., 2011; Latin America^7^	Survey	HIV programmes and patients	Data was collected on 90 stock-out events and 89 risk-of-stock-out episodes.	Zidovudine + Lamivudine, Abacavir + Lamivudine, Abacavir, Lamivudine, Saquinavir, Tenofovir, Lopinavir, Raltegravir, Fosamprenavir Calcium.
Pierre-Marie David 2014^18^	Qualitative study combining observant participation, ethnographic interviews, and focus groups	Patients and healthcare personnel	Data were gathered through participation in three clinics, interviews, and focus groups with community associations	First-line ARVs including nucleoside inhibitors (AZT/3TC, d4T/3TC) and non-nucleoside inhibitors (nevirapine, efavirenz).
Fokam et al., 2015; Cameroon^19^	Retrospective analysis of HIV Drug Resistance Early Warning Indicators (EWIs)	The study evaluated patients on ART across 15 sites.	15 ART sites	First and second line antiretrovirals (ARVs)
Nyogea et al., 2015; Tanzania^20^	Exploratory and cross-sectional study	HIV facilities and patients	12 healthcare sites surveyed, with structured interviews involving staff and patients.	Antiretrovirals (ARVs), co-trimoxazole, rapid diagnostic test kits for HIV.
Barnett et al., 2016; Malawi^21^	Cross-sectional study	Adult HIV-positive patients	92 confirmed cases	AVRs
Poku et al., 2017; Ghana^22^	A qualitative approach with semi-structured interviews	Women living with HIV (WLHIV)	55 participants, comprising 40 WLHIV and 15 professionals.	Specific antiretroviral drugs are not explicitly named in the study.
Brennan et al., 2017; South Africa^23^	A retrospective cohort study	HIV-positive individuals on ART	10,895 patients	Tenofovir disoproxil fumarate
Meloni et al., 2017; Nigeria^24^	A retrospective cross-sectional	Adults living with HIV	56 participants	Key antiretroviral drugs impacted by the shortages included stavudine (d4T) and zidovudine (AZT).
Moriarty et al., 2017; Ghana^25^	Qualitative study using semi-structured interviews.	Women living with HIV (WLHIV)	40 women living with HIV and 15 individuals from HIV-related projects/organizations.	Antiretroviral (ARV) drugs (specific names not detailed).
Dimala et al., 2017; Cameroon^26^	A retrospective cross-sectional study	HIV-positive individuals	100 patients	First-line ART drugs, including Tenofovir (TDF), Lamivudine (3TC), and Efavirenz (EFV)
Bajunirwe et al., 2018; Uganda^27^	Qualitative study using interviews and focus group	HIV-positive individuals, healthcare workers, and community stakeholders	Not explicitly mentioned but includes multiple stakeholder groups from 19 districts.	Antiretroviral drugs (ARVs), cotrimoxazole, and HIV test kits.
Tong et al., 2018; Cameroon^28^	Cross-sectional, mixed-methods survey	ART-treated patients	1885 ART-treated patients.	ART stock-outs but did not specify particular drug names.
Gils et al., 2018; Congo^9^	Cross-sectional survey	Facilities providing ART to people living with HIV (PLHIV).	92 facilities and 27 zonal warehouses.	Tenofovir-Lamivudine-Efavirenz (TDF-3TC-EFV); Zidovudine-Lamivudine-Nevirapine (AZT-3TC-NVP); Cotrimoxazole.
Hwang et al., 2019; South Africa^29^	A cross-sectional survey	Public health facilities providing ARV and TB treatment across South Africa.	2370 health facilities.	Adult ARVs: First-line (11%), second-line (15%), less commonly used (11%)- Pediatric ARVs: 9%- Nevirapine syrup for mother-to-child transmission: 1%- TB medicines: 4%.
Zakumumpa et al., 2019; Uganda^30^	Qualitative research design within a larger mixed-methods study	Health facility staff	76 semi-structured interviews	ARV medicines (specific drugs were not listed) specifically paediatric formulations.
Bernard et al., 2022; Kenya^31^	Cross-sectional telephone surveys	Women living with HIV (WLHIV)	784 (96%) completed the survey.	Stock-outs reported for HIV medications (mainly ART)
Jacobs et al., 2024; Kenya and Uganda^32^	Cross-sectional survey	Children living with HIV	144 health facilities (72 in Kenya, 72 in Uganda), including both public and private-not-for-profit facilities.	Dolutegravir (DTG), abacavir/lamivudine, lopinavir/ritonavir, nevirapine oral solution, and other limited-use ART drugs.
Rebecca Irons 2024; Venezuela^33^	Qualitative study based on interviews and testimonies	Individuals living with HIV in Venezuela and Venezuelan migrants in Colombia.	6 interviews with Venezuelan HIV activists (2024), supported by 40 testimonies from Venezuelan migrants living with HIV	Antiretroviral drugs (specific drugs not listed in the summary).
Sliefert et al., 2024; Kenya^34^	Qualitative study with focus groups (FGD) and key informant interviews (KII).	Caregivers and healthcare providers those managing paediatric HIV care.	72 caregivers (caring for 83 children) and 30 healthcare providers across 3 hospitals in Kenya.	Specific drugs were not mentioned in the study
Loung et al., 2025; Australia	Before and after	HIV patients on treatment and prevention	NA	Tenofovir and emtricitabine based formulation
Schmutz et al., 2026; Africa	Survey	HIV patients	172 HIV clinics	TB preventive regimens (INH based and Rifampicin based)
Lufesi et al., 2007; Malawi^35^	Cross-sectional audit and retrospective review	Patients seeking treatment malaria in the Lilongwe District, Malawi.	8 health centres (randomly selected from 37 in the district); 8968 patient records	Anti-malarial drugs: Quinine injections (9% supply), SP and para-aldehyde.
Njogu et al., 2008; Kenya^36^	Cross-sectional survey	Government health facilities and health workers	211 health facilities and 654 health workers.	Artemether-lumefantrine (AL) tablets (including different weight-specific packs).
Kangwana et al., 2009; Kenya^12^	Cross-sectional survey	Health facilities	164 health facilities (115 dispensaries, 30 health centres, 19 hospitals)	Artemether-lumefantrine (AL), particularly the weight-specific packs for younger children
Bogale et al., 2015; Ethiopia^37^	Qualitative study	Multi-level stakeholders, including policymakers, programme managers, advocacy groups, and healthcare service providers	49 stockholders	Chloroquine/primaquine shortages
Chuma et al., 2010; Kenya^38^	A mixed-methods study	The study focused on individuals and households	708 households participated in the survey	Artemisinin-based combination therapies (ACTs), the first-line treatment for malaria.
Alba et al., 2010; Tanzania^39^	A longitudinal observational design	Rural population and urban population	10 public health facilities	Sulphadoxine-pyrimethamine (SP) and artemether-lumefantrine (ALu)
Barrington et al., 2010; Tanzania^40^	The study was a 21-week pilot project (2009–2010) conducted across 129 health facilities in three rural districts in Tanzania. It used SMS technology and electronic mapping for drug supply management.	Rural health facilities	129 health facilities	Anti-malarial drugs, specifically artemisinin-based combination therapies (ACTs).
Windisch et al., 2011; Uganda^41^	A qualitative and observational study	People living with HIV/AIDS	Data from key informant interviews and health service delivery observations in a study district.	ART drugs, including antiretrovirals, in short supply due to supply chain inefficiencies.
Sudoi et al., 2012, Kenya^42^	18 cross-sectional surveys	Malaria patients	162–176 facilities	Artemether-lumefantrine (AL) in weight-specific packs: 6, 12, 18, and 24 tablets
Githinji et al., 2013; Kenya^43^	A cluster-randomized, interventional study	Health workers and facilities	87 public health facilities	Artemether-Lumefantrine (AL) in various formulations (6, 12, 18, 24), and Rapid Diagnostic Tests (RDTs).
Malik et al., 2013; Pakistan^44^	A qualitative study using semi-structured interviews.	Hospital pharmacists	16 hospital pharmacists	Antimalarial drugs (specific drugs not mentioned in the study, but antimalarials in general).
Mikkelsen-Lopez et al., 2013; Tanzania^45^	Retrospective study	333 households	333 respondents from Ulanga District.	Artemisinin-based Combination Therapy (ACT).
Chipwaza et al., 2014; Tanzania^46^	A qualitative study using 12 Focus Group Discussions and 14 in-depth interviews	Rural community members	93 community members participated in FGDs, and 14 health workers were interviewed.	Anti-malarial
Mikkelsen-Lopez et al., 2014; Tanzania^8^	A descriptive observational study	Public health facilities	Data collected from over 5000 public health facilities across Tanzania.	Artemisinin-based combination therapy (ACT), specifically artemether-lumefantrine, used for treating malaria.
Kabaghe et al., 2017; Malawi^47^	A cross-sectional survey	Guardians and children under 5 years presenting with symptoms suggestive of malaria	120 children and their guardians	Artemisinin-lumefantrine (first-line ACT for malaria).
Sirotich et al., 2020; Global^48^	Online survey	Patients with rheumatic diseases, including those in Africa, Southeast Asia, Americas, and Europe, who were prescribed antimalarials for their condition.	9393 respondents, of which 3872 (41.2%) were taking antimalarials.	Hydroxychloroquine, chloroquine.
Mengesha et al., 2021; Ethiopia^49^	Facility-based cross-sectional study using quantitative and qualitative methods (explanatory sequential mixed methods).	All public health facilities in Oromiya Special Zone, including 27 health centres and 2 hospitals; key informants: 12 health professionals managing anti-malaria pharmaceuticals.	Quantitative: 29 facilities. Qualitative: 12 key informants.	Artemether-lumefantrine, artesunate injection, primaquine tablets, chloroquine syrup, rapid diagnostic test (RDT).
Aberese-Ako et al., 2021; Ghana^50^	An ethnographic approach, combining non-participant observation with case studies, informal conversations, and in-depth interviews (IDIs).	The study focused on pregnant women receiving ANC in the Ashanti and Volta regions of Ghana, healthcare providers, and community members.	The study involved data collection from 8 health facilities and 8 communities.	Sulfadoxine-pyrimethamine (SP)
Tefera et al., 2022; Ethiopia^51^	Cross-sectional descriptive study	Patients receiving care at Shegaw Motta General Hospital and Motta Health Centre	The study included all the available essential medicines for a duration of 6 months at the two health facilities.	ART, Antimalarial and anti TB
Frosch et al., 2022; USA^52^	Cross-sectional survey	Individuals traveling from the USA to malaria-endemic regions, particularly immigrants, international travellers, and tourists.	Data from 21 retail pharmacies in Minneapolis/Saint Paul and New York City, surveyed between 2018 and 2020.	Atovaquone-proguanil, Artemether-lumefantrine, chloroquine, mefloquine, and doxycycline.
Capstick et al., 2011; UK^53^	Cross-sectional survey	TB patients treated at NHS centres, including children requiring liquid formulations and individuals with multidrug-resistant TB (MDR-TB).	77 treatment centres responded to the survey (46% response rate).	First-line TB drugs for drug-susceptible TB- Second-line drugs for MDR-TB- Licensed liquid formulations for paediatric patients.
Seaworth et al., 2013; USA^54^	A retrospective study	TB patients, including those with multidrug-resistant (MDR) and extensively drug-resistant (XDR) TB	Survey data covered approximately 81% of responding jurisdictions that reported MDR TB cases during the study period. Specific patient numbers were not provided.	Capreomycin, cycloserine, ethionamide, kanamycin, amikacin, and para-aminosalicylic acid.
Ballif et al., 2014; Global^55^	A cross-sectional study	HIV-infected adults with tuberculosis (TB), receiving ART in lower-income countries	Program-level data from 47 ART programs; Patient-level data from 1002 adult TB patients at 40 participating ART programs.	Second-line anti-TB drugs (specific drugs not detailed in the summary).
Singh et al., 2017; India^56^	Mixed-method study, including quantitative data collection and qualitative insights	Children (under 5 years) in Bhopal, India, who are contacts of smear-positive TB patients.	59 children, all living in households with at least one adult diagnosed with TB.	Isoniazid (100 mg Paediatric dose), the primary drug for preventive therapy.
Koomen et al., 2019; South Africa^57^	A cross-sectional study	TB patients	Data aggregated at the district level from health facilities reporting TB drug stockouts; sample included 51 districts (out of 52).	TB drugs (specifics not listed but encompassing drugs necessary for treatment regimens).
Sintayehu et al., 2022; Ethiopia^58^	A cross-sectional study using both qualitative and quantitative methods	Public health facilities	13 health facilities, with data from 67 health workers (including pharmacy managers and health officials).	Rifampicin, Isoniazid, Pyrazinamide, Ethambutol (first-line anti-TB drugs).
Otto-Knapp et al., 2024; Europe^59^	An observational and cross-sectional study	The study covers countries in the WHO European Region. Populations affected include patients diagnosed with MDR-TB and RR-TB, particularly those in resource-constrained settings.	The exact sample size is not specified, but the report includes data from several countries across the region, with a focus on the availability of essential drugs.	Bedaquiline, Delamanid, Linezolid, Clofazimine, and a few other second-line anti-TB drugs
Nabity et al., 2024; USA^60^	Retrospective survey	Local TB programs in California	61 local TB programs across California	First-line: Rifampin, Rifapentine, Rifabutin, Isoniazid, Pyrazinamide, Ethambutol; Second-line: Cycloserine, Ethionamide, Levofloxacin, Linezolid, Moxifloxacin, Para-aminosalicylate, Pretomanid.
Sreevidya et al., 2025, India^61^	Mixed method study	Kids with diagnosed pulmonary tuberculosis patients	96 kids	INH syrup children

The table summarises the study design, study population, sample size, and medicines reported to be affected by stock-outs across the included studies. Abbreviations: ACT: artemisinin-based combination therapy; AIDS: acquired immunodeficiency syndrome; AL: artemether–lumefantrine; Alu: artemether–lumefantrine; ANC: antenatal care; ART: antiretroviral therapy; ARV: antiretroviral; cART: combination antiretroviral therapy; d4T: stavudine; DTG: dolutegravir; EFV: efavirenz; FGD: focus group discussion; HIV: human immunodeficiency virus; IDIs: in-depth interviews; INH: isoniazid; KIIs: key informant interviews; MDR-TB: multidrug-resistant tuberculosis; NA: not available; NHS: National Health Service; PLHIV: people living with human immunodeficiency virus; RDT: rapid diagnostic test; RR-TB: rifampicin-resistant tuberculosis; SMS: short message service; SP: sulfadoxine–pyrimethamine; TB: tuberculosis; TDF: tenofovir disoproxil fumarate; WHO: World Health Organization; WLHIV: women living with human immunodeficiency virus; XDR-TB: extensively drug-resistant tuberculosis; 3TC: lamivudine; AZT: zidovudine (azidothymidine); ZDV: zidovudine.

**Table 2:** Distribution of the geographical location of studies selected in the systematic review.

Continent	Country	Income status	Number
Africa	Ethiopia	LIC	4
	Ghana	LMIC	3
	Cameroon	LMIC	4
	Uganda	LMIC	6
	Cote d’ivoire	LMIC	1
	Malawi	LIC	3
	Kenya	LMIC	7
	South Africa	UMIC	4
	Nigeria	LMIC	1
	Central Africa		1
	Tanzania	LMIC	6
	Democratic Republic of the Congo	LIC	1
	Venezuela	LMIC	1
Asia	India	LMIC	2
	Pakistan	LMIC	1
Europe	Serbia & Montenegro	UMIC	1
	UK	HIC	1
	Europe	HIC	1
North America	USA	HIC	2
Australia	Australia	HIC	1
South America	Latin America		1
	Brazil	UMIC	1
Global	Global		3

The table summarises the number of included studies by continent, country, and World Bank income classification. Abbreviations: HIC: high-income country; LIC: low-income country; LMIC: lower-middle-income country; UMIC: upper-middle-income country.

**Table 3:** Study design of the included studies.

Study design	Number of studies (n)
Mixed method (n = 29)	
Cross-sectional survey	12
Cross-sectional study	7
Interview based Survey	5
Other	5
Quantitative (n = 10)	
Cross-sectional study	3
Cross-sectional questionnaire-based survey	1
Interrupted Time series analysis	1
Survey	1
Retrospective cohort study	4
Qualitative (n = 12)	
Interview and group discussion based	10
Survey	2
Others (n = 5)	
Interview based ethnographic studies	2
Interventional study	1
Unknown	2

Despite differences in context, most studies consistently reported on three core aspects: (1) the proximate and systemic causes of drug shortages/stock-outs (n = 39); (2) the clinical and economic impacts of these stock-outs on patients and health systems (n = 41); and (3) the mitigation strategies either implemented during the stock-out or recommended to prevent future occurrences (n = 36). Below, we synthesize findings across all three disease areas according to these themes. Key insights are summarized in (Table 1; Supplementary Table S2).

### Causes of shortages and stock-outs

**Fig. 2:**
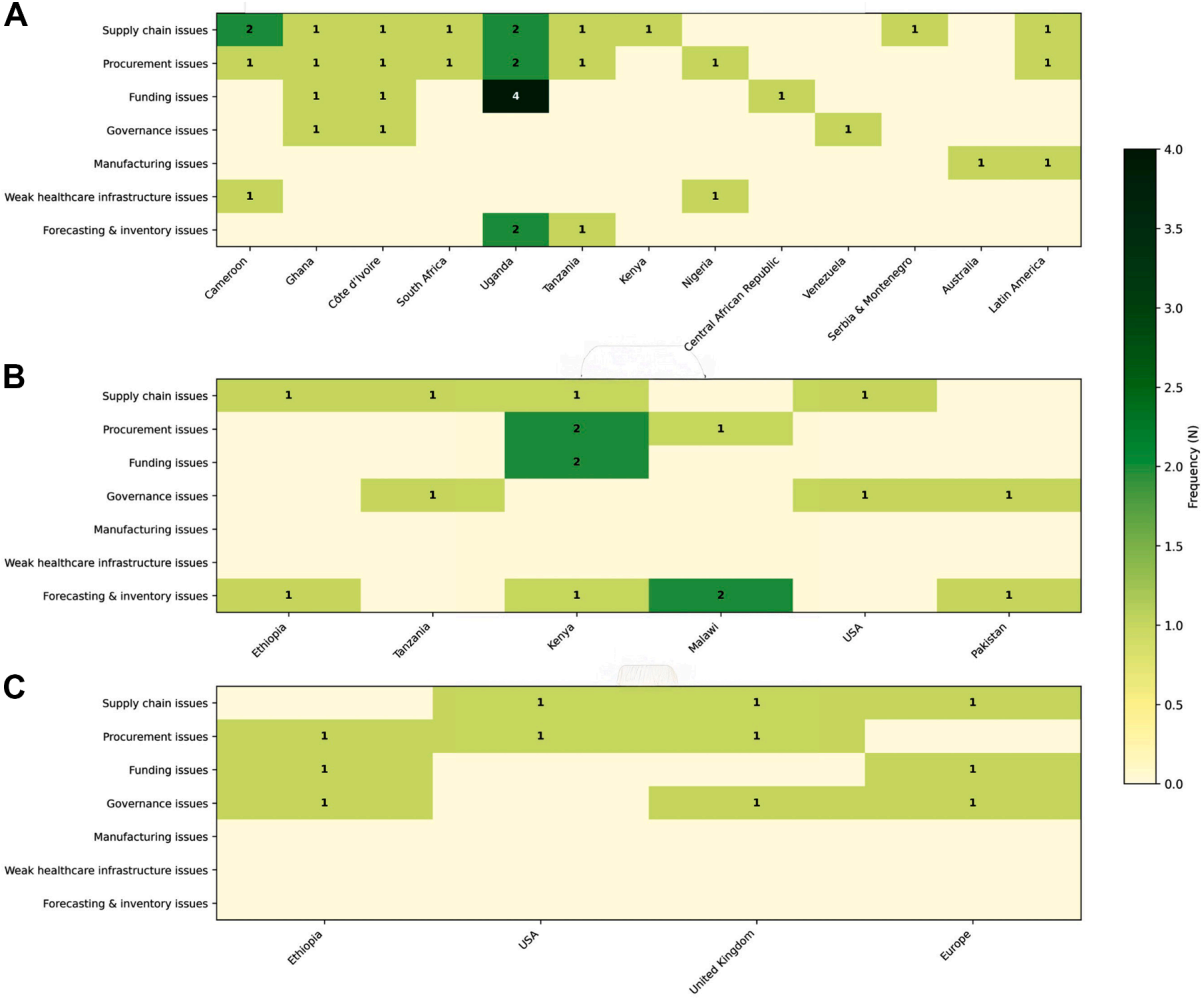
**Distribution of reported causes of stock-outs by region and disease category.** Heatmap showing the frequency of reported causes of medicine stock-outs across different geographic regions and disease categories. The main reasons for stock-outs include supply chain issues, procurement challenges, funding constraints, governance factors, manufacturing issues, healthcare infrastructure limitations, and forecasting and inventory problems. The panel represents HIV (A), malaria (B) and tuberculosis (C).

A complex interplay of factors was found to contribute to medicine stock-outs in HIV, tuberculosis and malaria programmes. Fig. 2 presents an overview of the most commonly reported causes of stock-outs, highlighting supply chain and logistics challenges as the predominant contributors, followed by procurement inefficiencies and financing-related constraints (additional data in Supplementary Table S3).

### Supply chain and inventory management issues

A number of studies (n = 28) identified weaknesses in supply chain infrastructure at both national and facility levels as key contributors to stock-outs. Problems included poor inventory management (failure to accurately track stock levels), insufficient logistic and storage capacity at facility level and limited coordination and distribution bottlenecks at national level. In Ghana, poor inventory management and distribution delays drove antiretroviral stock-outs^22^ while in Malawi^35^^,^^47^ and Uganda,^41^ record–supply discrepancies caused shortages. Limited transport to remote clinics were another contributing factor. In Serbia’s transition economy, a generally “weak supply chain” could not reliably get antiretrovirals to patients.^15^ These issues caused uneven distribution, with some facilities running out while others held excess stock, indicating poor redistribution. A lack of communication or data flow in the supply chain made stakeholders unaware of looming shortages until stock was exhausted.

### Procurement and regulatory delays

Studies (n = 17) also reported procurement process failures as a root cause. This encompassed delays and inefficiencies in tendering, bidding, and purchasing of medicines. In a Pan-American survey, delays in the bidding process were the single most common reason for antiretroviral supply gaps, accounting for 29% of at-risk-of-stock-out episodes.^7^ Likewise in Kenya, confusion over a new tendering process and lack of timely international procurement led to antimalarial stock-outs when policy switched to artemisinin-based therapy.^12^^,^^38^ International procurement can introduce vulnerabilities: shipping and importation delays were blamed for 19% of Latin American antiretroviral stock-out events^7^ and 71% of U.S. tuberculosis second-line medicine interruptions. Some specialised medicines such as these tuberculosis medicines required complicated regulatory procedures (e.g., importation under investigational drug protocols), which hampered rapid replenishment.^54^ Manufacturing problems also fell in this category e.g., global production shortages or supplier issues^7^ noted in the U.S. and European reports.

### Financing and budget issues

Sixteen studies identified funding shortfalls or financial mismanagement as a cause of stock-outs. This is especially evident in settings reliant on external donors. For instance, Uganda’s national antiretroviral program experienced a major stock-out in 2009 after a Global Fund aid shortfall, leaving the country without enough antiretrovirals and resulting in patient deaths.^17^ Delays in disbursement of government or donor funds were reported to postpone medicine orders e.g., 9% of Latin American cases were due to delayed budget allocation.^7^ In Cameroon and Central African Republic, corruption and mismanagement led to donors (Global Fund) freezing grants, which directly precipitated national antiretrovirals shortages.^18^ Even when funds were available, bureaucratic red tape in releasing money to purchase medicines caused bottlenecks.^8^ Several studies noted that reliance on international donors leaves countries without strong domestic funding and unable to pay suppliers if donor support is reduced or delayed.^8^^,^^16^^,^^18^^,^^22^^,^^27^^,^^41^ In Pakistan, inadequate hospital budgets for essential medicines and lack of enforcement of an essential medicines list meant frequent stock-outs of even inexpensive medications.^44^

### Governance and policy factors

Beyond funding issues, eight studies highlighted administrative and governance issues including a lack of oversight and poor system design for stock-outs to occur and persist^11^ In Tanzania, bureaucratic hurdles caused delays within donor agencies and country systems level antimalarial supply.^8^^,^^11^ In Ghana, weak coordination e.g., port clearance delays due to miscommunication with customs contributed to antiretroviral delivery holdups.^22^ Some national programmes lacked clear guidelines or contingency plans for maintaining medicine stock levels. In the United Kingdom (UK), the absence of national guidance on stock management meant each treatment centre working in silo, leading to inconsistent supplies across regions.^53^ Governance challenges also appeared in the form of centralisation: in the Central African Republic, Global Fund–supported free antiretrovirals created a single supply system, and when programme funding was frozen officially for poor performance but widely attributed to corruption the entire system collapsed, leaving patients without alternatives.^18^

### Poor planning and forecasting

Inadequate forecasting or planning as a cause were explicitly cited in six studies, though this factor underlies many supply chain failures. Rapid program expansions or policy changes without proper planning featured here. For instance, Nigeria’s rapid antiretroviral therapy (ART) scale-up outpaced procurement planning, resulting in antiretroviral shortages shortly after expansion.^24^ Similarly, when Tanzania changed its malaria treatment policy, many facilities were unprepared for the transition, resulting in sulfadoxine/pyrimethamine running out before artemether–lumefantrine combination was widely distributed.^39^ Several studies noted a lack of reliable consumption data or forecasting tools, meaning orders were not aligned with actual needs.^19^^,^^30^^,^^32^^,^^49^^,^^58^ In Ethiopia, failure to use available tools (like look-ahead seasonality indices for malaria) led to understocking before high-demand seasons.^49^ In summary, stock-outs often reflected a failure to accurately predict demand due to data gaps or sudden changes, and to plan procurement accordingly.

### Demand spikes and external shocks

Several studies reported that stock-outs were precipitated by unexpected increases in demand or other external crises. In many settings, health programmes experienced patient volumes that exceeded initial projections. For example, in Latin American programmes reported 10% of stock-out episodes were due to unexpectedly high patient enrolment or usage of health services.^7^ Seasonal variation also contributed to demand surges; in malaria-endemic regions, increased transmission during the rainy season led to higher medicine requirements, and where supply systems failed to anticipate these patterns, stock-outs occurred.^49^ While these factors are partly out of the control of supply managers, resilient systems require buffer stocks or surge capacity to handle demand shocks. External crises further compound these challenges. For instance, during the Ebola outbreak and Corona virus disease of 2019 (COVID-19) pandemic, repurposing of antimalarial drugs such as hydroxychloroquine led to shortages for patients requiring them for approved indications in malaria. In addition, the COVID-19 pandemic exacerbated existing vulnerabilities in global supply chains, further increasing the risk of medicine stock-outs.^48^

### Health system infrastructure and human resources

A few studies noted contributory factors such as limited human resources or infrastructure for supply management including clinics with no dedicated pharmacy staff to manage orders, leading to inconsistent stock management. In Ethiopia, insufficient logistics training among staff contributed to poor stock handling and errors in ordering.^49^ In Cameroon, rural clinics faced stock-outs partly due to limited storage and transport capacity, as well as staff shortages, making it hard to maintain buffer stocks locally.^19^ These capacity issues are often intertwined with other categories (e.g., supply chain problems can stem from not having trained supply chain managers). Strengthening the health workforce and infrastructure was thus implied as a preventive measure.

### Prescribing and usage factors

Although less frequently cited, a few reports pointed to inappropriate medicine use or prescribing practices. In Pakistan, irrational prescribing of antimalarials (e.g., overuse or non-adherence to treatment guidelines) and lack of generic substitution, strained limited supplies caused frequent shortages.^44^ In Malawi, over-prescription and wastage were observed alongside reporting delays, leading to stock imbalances.^47^ Patient behaviour can also play a role: one study from Cameroon suggested that delays in patients picking up refills in rural areas led to unpredictable inventory levels and ultimately stock-outs when many came at once or drugs expired on shelf.^19^

### Impact of drug stock-outs

**Fig. 3:**
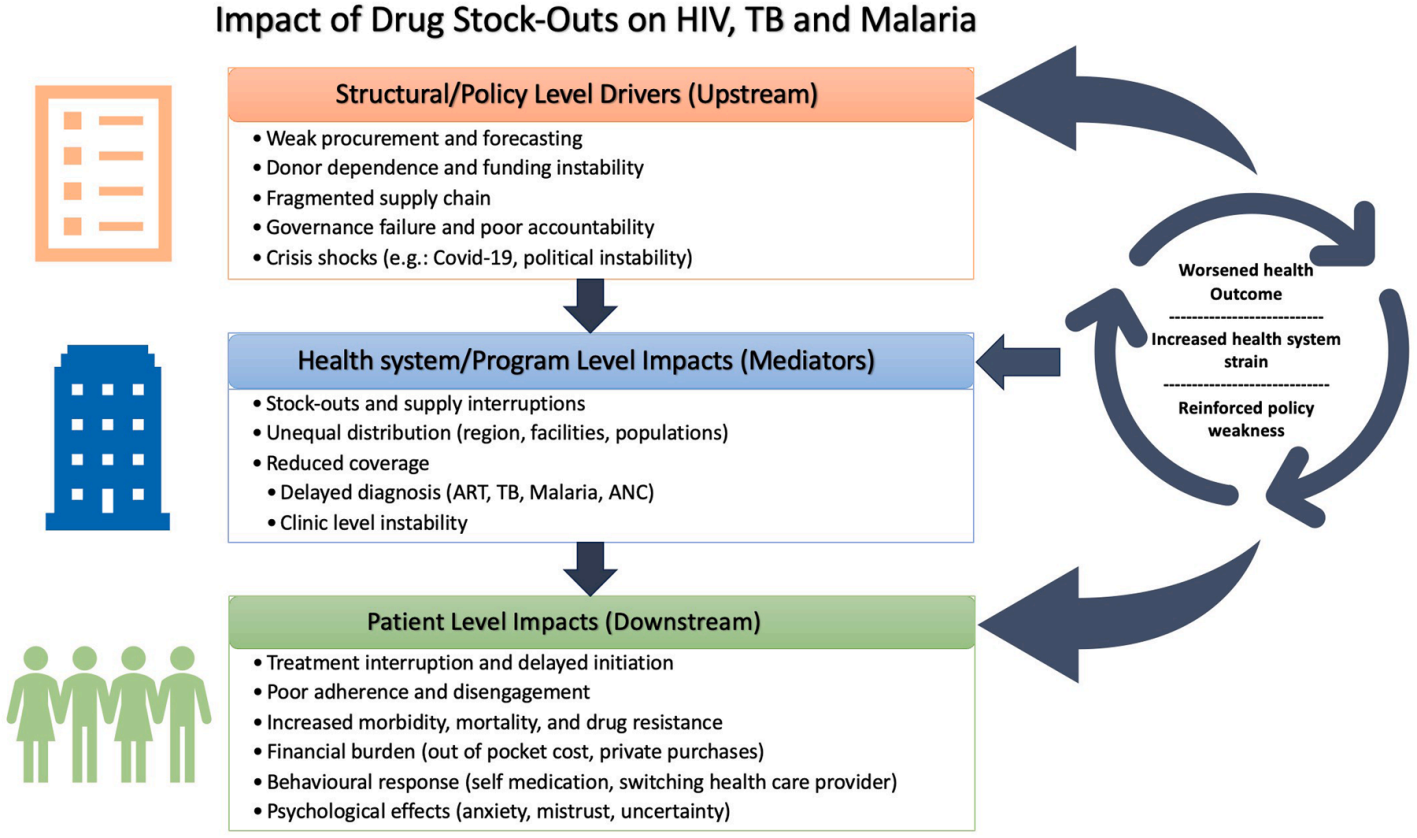
**Conceptual framework of drivers and impacts of drug stock-outs.** Conceptual framework illustrating the upstream structural and policy-level drivers (e.g., weak procurement, funding instability), intermediary health system and program-level impacts (e.g., stock-outs, coping strategies), and downstream patient-level consequences (e.g., treatment interruption, increased morbidity, financial burden, and psychosocial effects). The framework highlights the cyclical relationship between system weaknesses and adverse health outcomes. HIV: Human Immunodeficiency Virus; TB: Tuberculosis; ART: Antiretroviral Therapy; ANC: Antenatal Care; COVID-19: Coronavirus Disease 2019.

All included studies underscored that medication stock-outs have negative consequences for patient care and health outcomes. The multilevel conceptual framework highlights structural weaknesses at the policy level such as procurement inefficiencies, donor dependence, and governance failures, drive program-level disruptions leading to stock-outs, weak logistics, and compromised service delivery. These disruptions translate into patient-level consequences-treatment interruptions, poor adherence, increased costs, and adverse health outcomes. Reinforcing feedback loops further exacerbate system inefficiencies, perpetuating shortages and worsening outcomes across HIV, tuberculosis, and malaria (Fig. 3). Importantly, these interconnected effects extend to clinical consequences and impose a substantial economic burden on both patients and health systems.

### Clinical impact

A consistent finding was that medicine stock-outs lead to unplanned treatment interruptions, which jeopardizes disease control at both individual and population levels.^11^^,^^17^^,^^22^^,^^25^ In HIV programs, even short interruptions in antiretrovirals can result in viral rebound and increased risk of HIV progression or opportunistic infections.^7^^,^^17^^,^^19^^,^^22^ Many studies reported that stock-outs forced patients to miss doses or stop treatment entirely for some period.^7^^,^^11^^,^^19^ For example, a large cohort in Abidjan, Côte d’Ivoire found that 11% of patients initiating antiretrovirals experienced a treatment gap or regimen change due to stock-outs, and those who discontinued therapy had significantly higher mortality.^11^ Qualitative studies from Ghana and Uganda described patients’ distress when life-saving medicines were suddenly unavailable, sometimes causing them to ration pills or skip doses, with some assuming personal blame for “non-adherence” when in fact the system failed to supply medicines.^22^

Interruptions or suboptimal regimens have been linked to drug-resistance. In Nigeria, patients affected by antiretroviral stock-outs showed a higher frequency of HIV drug resistance mutations (e.g., thymidine analogue mutations) upon re-supplying, compared to those without interruptions.^24^ In Cameroon, treatment gaps were a significant predictor of virologic failure and resistance development.^19^ During nationwide stock shortages, the UK reported Multi-Drug Resistant (MDR)-tuberculosis treatment interruptions in 16% of cases, risking amplified resistance.^53^ Similarly, in the USA patients were placed on less effective regimens, increasing the risk of treatment failure and further resistance.^54^^,^^60^

Studies from tuberculosis, HIV, and malaria programs have documented shortages and stock-outs globally and linked them to lower treatment success, higher mortality, treatment failure, disease progression, and increased transmission.^12^^,^^15^^,^^16^^,^^18^^,^^22^^,^^23^^,^^28^^,^^31^^,^^44^^,^^45^^,^^49^^,^^56^^,^^57^ Clinical Impacts for most of the shortages or stock-outs include missed clinic visits, clinical deterioration, opportunistic infections, and use of less effective drugs. Psychological effects such as anxiety and loss of confidence in health systems are also reported.^15^^,^^18^^,^^22^^,^^23^^,^^28^

### Economic impact

Studies highlight the burden to patients when public-sector facilities run out of free medicines, forcing them to seek care in the private sector or from distant clinics, leading to high out-of-pocket expenses and travel costs.^20^^,^^22^^,^^23^^,^^31^^,^^45^ In Uganda, patients reported spending significant amounts on transport to other districts or on purchasing antiretrovirals from private pharmacies during stock-outs.^27^ In Tanzania, households faced increased out-of-pocket expenditure for fever treatment when public clinics lacked malaria medicines, sometimes resorting to less qualified drug shops that charged fees.^45^ For low-income patients, these expenses can be catastrophic, leading some to forgo treatment.

Healthcare systems themselves face inefficiencies and extra costs due to stock-outs. One major cost is emergency procurement.^7^^,^^12^^,^^58^^,^^59^ Emergency procurement including purchasing limited quantities urgently at higher prices is common; nearly half (46%) of antiretroviral stock out events in a Pan American Health Organisation survey triggered such purchases.^7^ In addition to system-level costs, stock-outs can shift the financial burden onto patients. For instance, a study from USA highlighted that antimalarial preventive treatment is not covered under Universal Health Coverage (UHC), which contributed to higher costs for prescribed medications compared with over-the-counter alternatives. Importantly, most reports emerged from LMICs where UHC is largely unimplemented across geographies. Additionally, if patients interrupt first-line therapy and subsequently require more expensive second-line medicines, as in Cameroon where HIV drug resistance would significantly raise antiretroviral costs.^17^^,^^19^^,^^24^

Stock-outs can also lead to wastage of resources because some facilities overstock medicines, leading to expirations. An Ethiopian study reported an 11.3% wastage rate of antimalarials in some facilities that had overstocked while others had none.^49^ Treating an MDR-tuberculosis case, which might have been prevented by continuous first-line therapy is far more costly for health services. One global report emphasized that weak tuberculosis medicine supply in HIV programs led to under-treatment of tuberculosis, which can result in increased long term healthcare costs.^59^ These include increased treatment costs due to the use of alternative drugs, higher hospitalisation rates, and potential long-term health system burdens from unmanaged tuberculosis cases.

At a macro level, stock-outs undermine health program sustainability and can harm economies, as seen in Venezuela’s antiretroviral treatment collapse, which contributed to broader health and productivity losses.^33^ Frequent shortages may deter funders and waste prior investments, while emergency donor shipments lead to unplanned cost.^41^ It was observed in Uganda’s antiretroviral crisis where emergency shipments supplied by donors were essentially unplanned expenditures that could have been avoided with proper initial funding and management.^16^

### Mitigation strategies and proposed solutions

The included studies and reports described a variety of strategies that were used to manage medicine shortages when they occurred, as well as longer-term solutions suggested to prevent future stock-outs. We differentiate between immediate coping mechanisms and broader system-level improvements, acknowledging that these often overlap (Box 1).

Box 1 **Mitigation strategies for drug shortages**

**Table:** 

Short-term coping mechanisms	System-level and long-term strategies
**Emergency procurement & redistribution:** Rapid purchase, donor emergency stock (e.g., Global Fund), borrowing between facilities/countries.^7^^,^^16^^,^^22^^,^^25^^,^^27^^,^^29^^,^^41^^,^^49^^,^^50^^,^^58^ **Rationing & prioritization:** Restricting new initiations; prioritizing high-risk groups (e.g., child tuberculosis contacts, pregnant women for malaria prophylaxis).^11^^,^^22^^,^^27^^,^^41^^,^^46^^,^^50^^,^^56^^,^^60^ **Switching regimens/formulations:** Substitution with alternative antiretrovirals or tuberculosis drugs, temporary dual/mono “holding” regimens, local compounding of paediatric doses.^7^^,^^9^^,^^11^^,^^20^, ^21^, ^22^, ^23^^,^^52^^,^^53^^,^^56^ **Referral to other sources:** Directing patients to other facilities, pharmacies, Non-Governmental Organisations (NGOs), or informal networks.^27^^,^^29^^,^^30^^,^^33^^,^^52^ **Technology-enabled monitoring:** SMS/e-reporting systems (e.g., SMS for Life) enabling rapid redistribution and early warning.^8^^,^^20^^,^^40^^,^^43^ **Community/patient coping:** Peer sharing of drugs; NGO/faith-based emergency distribution.^15^^,^^18^^,^^33^	**Strengthening procurement & supply chains:** Timely tendering, buffer stock, Logistics Management Information System, staff training, logistics infrastructure.^27^^,^^34^^,^^40^^,^^43^^,^^47^^,^^49^ **Diversifying supply & local/regional production:** Multiple suppliers, generics, pooled procurement (e.g., Pan American health Organisation Fund), inter-country borrowing agreements.^7^^,^^12^^,^^22^^,^^25^^,^^54^^,^^62^ **Sustainable financing & buffer stocks:** Increased domestic funding, ring-fenced budgets, national/regional reserves.^11^^,^^22^^,^^25^^,^^27^^,^^47^^,^^54^^,^^60^ **Policy & governance reforms:** Unified supply systems, central stock monitoring units, early warning systems, enforcement of Essential medicine list/Standard treatment guidelines, transparency and anti-corruption.^7^^,^^25^^,^^54^

While these strategies provide a comprehensive overview of responses to stock-outs, we also examined whether there were temporal trends in their underlying causes. Across studies from countries such as Tanzania,^8^^,^^39^^,^^40^^,^^46^ Kenya,^12^^,^^32^^,^^34^^,^^36^^,^^38^^,^^42^^,^^43^ and Ethiopia^37^^,^^51^^,^^58^ as well as across all included studies, no clear change in the drivers of stock-outs was observed over time. Key factors including procurement delays, weak supply chain systems, poor forecasting, and financing constraints were consistently reported across different periods. Even within the same country, the causes of stock-outs remained largely unchanged, suggesting persistent systemic challenges.

In contrast, the way stock-outs have been managed, has evolved over time. Earlier studies describe largely reactive approaches, where facilities relied on ad hoc measures such as borrowing medicines from nearby centres^41^ or placing urgent orders only after a stockout had already happened. Over time, particularly from early 2010s, more structured approaches began to emerge, including introduction of basic inventory management tools^8^ and basic logistics systems,^20^ although their implementation were inconsistent. By the late 2010s, management strategies became more coordinated, with improved communication across health system levels,^27^ enhanced staff training, and better use of data for decision-making.^23^ More recent evidence, particularly from studies conducted after 2020, indicates a shift towards proactive and resilience-focused strategies. These include the use of digital monitoring systems, maintenance of buffer stocks,^60^ and more strategic responses to large-scale disruptions. Overall, the findings suggest a gradual transition from short-term, reactive responses to more planned, coordinated, and system-oriented approaches to managing medicine shortages.

## Discussion

This review brings together evidence to draw a comprehensive picture of medicine stock-outs in three major infectious disease programmes. Despite the distinct characteristics of HIV, tuberculosis, and malaria programmes, our findings reveal commonalities in the drivers and consequences of medicine stock-outs across these diseases. Evidence from empirical studies identified that stock-outs predominantly stem from health system and supply chain shortcomings – notably, procurement delays, distribution failures, forecasting errors, and resource constraints. These findings were supported across multiple settings, although the strength of evidence varied, with some insights like worsened patient outcomes, increased burdens on both patients and health services were derived from observational studies and other programmatic reports.

One important insight is that the challenges are largely systemic and not disease-specific. For example, whether a stock-out involves antiretrovirals or antimalarials, the root cause might be a poorly functioning central medical stores or an inefficient procurement bureaucracy.^8^ This implies that strengthening the overall pharmaceutical supply chain in a country will yield benefits across multiple disease programmes.^63^ Vertically, disease-specific programmes have historically operated separate supply chains (especially HIV and malaria programmes heavily supported by global initiatives), but there is growing recognition that an integrated approach may improve resilience and efficiency.^64^ Integration, however, must be done carefully. HIV, tuberculosis, and malaria medicines each have unique storage needs, demand patterns, and distribution considerations. A balanced approach is needed: leveraging integration for common functions (like procurement, warehousing) while retaining some disease-specific expertise (for forecasting and regimen-specific supply management). However, a key limitation identified is the lack of reliable consumption data, which can hinder accurate forecasting. While the use of morbidity-based data (e.g., disease burden or notified cases) has been suggested as a potential alternative, it is not without challenges, including concerns around data quality, assumptions related to treatment guidelines and patient adherence, standardisation of reporting, and administrative burden. Notably, none of the studies included in this review reported the use of morbidity-based approaches for forecasting.^65^

Our review also highlights some unique challenges and considerations within each infection area. For HIV programmes, one challenge is the continual evolution of treatment guidelines and introduction of new formulations (such as new paediatric antiretroviral formulations, long-acting injectables, etc.).^66^^,^^67^ These advances, while beneficial clinically, can strain supply chains that must rapidly adapt as seen with the rollout of new first-line regimens causing transient stock-outs when supply lagged demand.^9^ Malaria programmes face additional challenge of seasonality, where medicine demand can fluctuate significantly, requiring careful stock management to meet peak needs while avoiding expiry during periods of lower demand.^30^^,^^38^Findings from this review also indicated that financing constraints are an important contributor to medicine stock-outs, although the strength of evidence varies across settings. Several included studies identified funding shortfalls, delayed disbursements, and financial mismanagement as direct causes of stock-outs, particularly in countries heavily reliant on external donor support. For example, HIV programmes also often have multiple funding sources and parallel supply systems (government, donor, etc.), making coordination crucial.^68^ On the other hand, tuberculosis programmes, especially for MDR-tuberculosis, are hindered by the limited global production of certain specialised drugs. Even if financing is available, there may be only a few suppliers internationally, which increases vulnerability to stock-outs if any production issue arises. Additionally, tuberculosis treatment is long and complex, so even a short stock-out can disrupt a carefully planned regimen.^69^

The clinical stakes of preventing stock-outs cannot be overstated. For HIV, consistent medicine supply is fundamental to achieving the UNAIDS 95-95-95 targets (means 95% of people living with HIV know their HIV status, 95% of people who know their status are receiving HIV treatment, and 95% of people on treatment are virally suppressed).^70^ Regular stock-outs directly jeopardize these targets by causing viral rebound and possible resistance. Similarly, for tuberculosis, any interruption in medicine supply threatens the End tuberculosis strategy goals, risking a resurgence of cases and drug-resistant strains which are difficult and expensive to treat.^54^ In malaria, stock-outs can undermine years of progress in reducing mortality; a single season of widespread antimalarial stock-outs could precipitate a spike in malaria deaths and cases, reversing declining trends. Addressing stock-outs is an essential component of disease control and elimination strategies. It is not merely an operational issue but one that impacts disease prevalence and mortality at the population level.^48^

Encouragingly, the review found evidence that targeted interventions can make a difference. Simple innovations like text message reporting, better training for staff, and improved coordination have reduced stock-outs in multiple contexts.^43^ For example, an important innovation in short-term stock-out management was leveraging technology (mobile phones, SMS, electronic reporting) to quickly identify and respond to supply gaps. The “SMS for Life initiative” in Kenya^43^ and Tanzania^8^ set up weekly text-message reporting of malaria drug stock levels from health facilities to a central database, allowing rapid redistribution and resupply to facilities running low. Similarly, one global recommendation for tuberculosis medicines was early notification to regulatory bodies (like the FDA) and to providers when a supply issue is anticipated.^54^ Having real-time visibility of stock levels via mHealth or electronic logistics management systems was frequently mentioned as a solution to prevent a local stock-out from becoming a crises.^20^ These success stories should be scaled up and adapted elsewhere. Moreover, global health stakeholders are increasingly aware of medicine stock-outs; initiatives by World Health Organisation (WHO) and others to define, measure, and publicly report stock-outs are steps in the right direction. WHO’s Health Emergencies programme now often monitors essential medicine availability during crises, and the Global Fund has built supply-chain metrics (including stock-out rates) into grant performance indicators. Such monitoring increases accountability and keeps the issue on the policy agenda.

Many studies in our review were observational or cross-sectional, providing snapshots of stock-out situations without necessarily quantifying the long-term impact. There is a paucity of rigorous quantitative research directly linking stock-outs to patient outcomes, especially for tuberculosis and malaria. While HIV literature provides comparatively more evidence,^11^ further studies are needed to evaluate the outcomes of patients through stock-out periods versus stable supply periods. Another gap is economic analysis: very few studies robustly quantified the economic cost of stock-outs (either to patients or health systems). Such analyses could strengthen the case for investing in supply chain improvements by demonstrating the cost-effectiveness of prevention (i.e., avoiding the much higher costs of emergency response and treating complications). In addition, this review synthesised evidence from qualitative, quantitative, and mixed-methods studies, findings from studies with varying levels of rigor were considered together, which may influence the strength and generalisability of the conclusions. However, given the nature of the research question, experimental designs such as randomised controlled trials are unlikely to be feasible, and real-world evidence from qualitative studies, observational data, and programmatic reports remains essential for understanding these system-level challenges.

Medicine stock-outs of HIV, tuberculosis, and malaria programs remain a persistent barrier to effective disease control in many parts of the world. This systematic review demonstrates that the causes of these stock-outs are largely preventable, rooted in modifiable supply chain and health system factors. Consequently, the solutions lie in fortifying systems – through better planning, robust logistics, sufficient financing, and innovative monitoring – rather than in biomedical advances alone. Addressing medicine stock-outs will yield cross-cutting benefits: more consistent treatment for patients, prevention of drug resistance, and greater progress toward global targets in HIV, tuberculosis, and malaria. It requires commitment from policymakers, donors, and health program managers alike to prioritize supply chain resilience. Ultimately, the true measure of success in global infectious disease programs will not only be the efficacy of the drugs we develop, but also our ability to deliver those medicines uninterrupted to the people who need them.

## Contributors

AKP, RKB, NS, and EC conceptualised and designed the study methodology. NS and EC secured funding for the study. AKP was responsible for data curation. AKP and RKB conducted the formal analyses and investigation. AKP, RKB, NS, and EC prepared the original draft of the manuscript. AKP, RKB, EC, JC, AG, AGM, UG, SD, MMil, SM, TT, SS, Mmen and NS contributed to the critical review and revision of the manuscript. NS and EC provided overall supervision of the study. AKP and RKB accessed and verified the underlying data. All authors had full access to all the data in the study, accepted responsibility to submit for publication, and read and approved the final version of the manuscript.

## Data sharing statement

The data are reported in previously published studies and accessible in corresponding publications.

## Declaration of interests

Thomas Tängdén serves as Chair of the Swedish Strategic Programme against Antibiotic Resistance (STRAMA). Financial support from the Swedish government is provided to his employer to cover salary costs associated with this role. Sanjeev Singh is supported by the Wellcome Trust (grant number 226730/Z/22/Z). Marc Mendelson is supported by the Wellcome Trust (grant number 226690/Z/22/Z). Esmita Charani is supported by the Global Antibiotic Research and Development Partnership (GARDP). The other authors declare no competing interests.

## References

[1] National Academies of Sciences, Engineering, and Medicine, Health and Medicine Division, Board on Global Health, Committee on Global Health and the Future of the United States (2017). http://www.ncbi.nlm.nih.gov/books/NBK458474/.

[2] Protecting key populations from abrupt disruptions to essential HIV services. https://www.who.int/news/item/27-02-2025-protecting-key-populations-from-abrupt-disruptions-to-essential-hiv-services.

[3] Strengthening supply chain resilience to safeguard health in Low- and middle-income countries - FP analytics. https://fpanalytics.foreignpolicy.com/2023/04/14/strengthening-supply-chain-resilience-to-safeguard-health-in-low-and-middle-income-countries/.

[4] Harries A.D., Schouten E.J., Makombe S.D. (2007). Ensuring uninterrupted supplies of antiretroviral drugs in resource-poor settings: an example from Malawi. Bull World Health Organ.

[5] Chanda-Kapata P., Ntoumi F., Kapata N. (2022). Tuberculosis, HIV/AIDS and malaria health services in sub-Saharan Africa – a situation analysis of the disruptions and impact of the COVID-19 pandemic. Int J Infect Dis.

[6] Trump order set to halt supply of HIV, malaria drugs to poor countries, sources say | Reuters. https://www.reuters.com/business/healthcare-pharmaceuticals/trump-order-set-halt-supply-hiv-malaria-drugs-poor-countries-sources-say-2025-01-28/.

[7] Sued O., Schreiber C., Girón N., Ghidinelli M. (2011). HIV drug and supply stock-outs in Latin America. Lancet Infect Dis.

[8] Mikkelsen-Lopez I., Shango W., Barrington J., Ziegler R., Smith T., deSavigny D. (2014). The challenge to avoid anti-malarial medicine stock-outs in an era of funding partners: the case of Tanzania. Malar J.

[9] Gils T., Bossard C., Verdonck K. (2018). Stockouts of HIV commodities in public health facilities in Kinshasa: barriers to end HIV. PLoS One.

[10] Bam L., McLaren Z.M., Coetzee E., Von Leipzig K.H. (2017). Reducing stock-outs of essential tuberculosis medicines: a system dynamics modelling approach to supply chain management. Health Pol Plann.

[11] Pasquet A., Messou E., Gabillard D. (2010). Impact of drug stock-outs on death and retention to care among HIV-Infected patients on combination antiretroviral therapy in Abidjan, Côte d’Ivoire. PLoS One.

[12] Kangwana B.B., Njogu J., Wasunna B. (2009). Malaria drug shortages in Kenya: a major failure to provide access to effective treatment. Am J Trop Med Hyg.

[13] Carmody E.R., Diaz T., Starling P., Santos A.P.R.B.D., Sacks H.S. (2003). An evaluation of antiretroviral HIV/AIDS treatment in a Rio de Janeiro public clinic. Trop Med Int Health.

[14] Marcellin F., Boyer S., Protopopescu C. (2008). Determinants of unplanned antiretroviral treatment interruptions among people living with HIV in Yaoundé, Cameroon (EVAL survey, ANRS 12-116). Trop Med Int Health.

[15] Bernays S., Rhodes T. (2009). Experiencing uncertain HIV treatment delivery in a transitional setting: qualitative study. AIDS Care.

[16] Wasswa H. (2009).

[17] Muhamadi L., Nsabagasani X., Tumwesigye M.N. (2010). Inadequate pre-antiretroviral care, stock-out of antiretroviral drugs and stigma: policy challenges/bottlenecks to the new WHO recommendations for earlier initiation of antiretroviral therapy (CD<350 cells/μL) in eastern Uganda. Health Policy.

[18] David P.M. (2014). Towards the embodiment of biosocial resistance? How to account for the unexpected effects of antiretroviral scale-up in the Central African Republic. Glob Public Health.

[19] Fokam J., Elat J.B.N., Billong S.C. (2015). Monitoring HIV drug resistance early warning indicators in Cameroon: a study following the revised world Health Organization recommendations. PLoS One.

[20] Nyogea D.S., Said H., Mwaigomole G. (2015).

[21] Barnett B., Chaweza T., Tweya H., Ngambi W., Phiri S., Hosseinipour M. (2016). Assessment of non-standard HIV antiretroviral therapy regimens at Lighthouse Trust in Lilongwe, Malawi. Mal Med J.

[22] Poku R.A., Owusu A.Y., Mullen P.D., Markham C., McCurdy S.A. (2017). HIV antiretroviral medication stock-outs in Ghana: contributors and consequences. Afr J AIDS Res.

[23] Brennan A.T., Bor J., Davies M. (2017). Tenofovir stock shortages have limited impact on clinic- and patient-level HIV treatment outcomes in public sector clinics in South Africa. Trop Med Int Health.

[24] Meloni S.T., Chaplin B., Idoko J. (2017). Drug resistance patterns following pharmacy stock shortage in Nigerian antiretroviral treatment program. AIDS Res Ther.

[25] Moriarty K., Genberg B., Norman B., Reece R. (2018). The effect of antiretroviral stock-outs on medication adherence among patients living with HIV in Ghana: a qualitative Study. J Assoc Nurs AIDS Care.

[26] Dimala C.A., Bechem N.N., Aroke D., Kadia B.M. (2017). Motives for change of first-line antiretroviral therapy regimens in an unselected cohort of HIV/AIDS patients at a major referral centre in South-west Cameroon. BMC Res Notes.

[27] Bajunirwe F., Tumwebaze F., Akakimpa D., Kityo C., Mugyenyi P., Abongomera G. (2018). Towards 90-90-90 target: factors influencing availability, access, and utilization of HIV services—A qualitative study in 19 Ugandan districts. BioMed Res Int.

[28] Tong C., Suzan-Monti M., Sagaon-Teyssier L. (2018). Treatment interruption in HIV -positive patients followed up in Cameroon’s antiretroviral treatment programme: individual and health care supply-related factors (ANRS -12288 EVOLC am survey). Trop Med Int Health.

[29] Hwang B., Shroufi A., Gils T. (2019). Stock-outs of antiretroviral and tuberculosis medicines in South Africa: a national cross-sectional survey. PLoS One.

[30] Zakumumpa H., Kiweewa F.M., Khuluza F., Kitutu F.E. (2019). “The number of clients is increasing but the supplies are reducing”: provider strategies for responding to chronic antiretroviral (ARV) medicines stock-outs in resource-limited settings: a qualitative study from Uganda. BMC Health Serv Res.

[31] Bernard C., Hassan S.A., Humphrey J. (2022). Impacts of the COVID-19 pandemic on access to HIV and reproductive health care among women living with HIV (WLHIV) in Western Kenya: a mixed methods analysis. Front Glob Womens Health.

[32] Jacobs T.G., Okemo D., Ssebagereka A. (2024). Availability and stock-outs of paediatric antiretroviral treatment formulations at health facilities in Kenya and Uganda. HIV Med.

[33] Irons R. (2024). Resistance and the regimen: the microthanatopolitics of Venezuelan antiretroviral scarcity and HIV drug adherence failures. Soc Sci Med.

[34] Sliefert M., Maloba M., Wexler C. (2024). Challenges with pediatric antiretroviral therapy administration: qualitative perspectives from caregivers and HIV providers in Kenya. PLoS One.

[35] Lufesi N.N., Andrew M., Aursnes I. (2007). Deficient supplies of drugs for life threatening diseases in an African community. BMC Health Serv Res.

[36] Njogu J., Akhwale W., Hamer D., Zurovac D. (2008). Health facility and health worker readiness to deliver new national treatment policy for malaria in Kenya. E Af Med Jrnl.

[37] Bogale K.A., Yenesew M.A., Alemu K., Muchie K.F., Asemahagn M.A., Enbiale W. (2025). Facilitators and barriers of malaria prevention and treatment services to pregnant women in Ethiopia: a multi-level health system analysis, 2025. Malar J.

[38] Chuma J., Okungu V., Molyneux C. (2010). Barriers to prompt and effective malaria treatment among the poorest population in Kenya. Malar J.

[39] Alba S., Hetzel M.W., Goodman C. (2010). Improvements in access to malaria treatment in Tanzania after switch to artemisinin combination therapy and the introduction of accredited drug dispensing outlets - a provider perspective. Malar J.

[40] Barrington J., Wereko-Brobby O., Ward P., Mwafongo W., Kungulwe S. (2010). SMS for life: a pilot project to improve anti-malarial drug supply management in rural Tanzania using standard technology. Malar J.

[41] Windisch R., Waiswa P., Neuhann F., Scheibe F., De Savigny D. (2011). Scaling up antiretroviral therapy in Uganda: using supply chain management to appraise health systems strengthening. Global Health.

[42] Sudoi R.K., Githinji S., Nyandigisi A., Muturi A., Snow R.W., Zurovac D. (2012). The magnitude and trend of artemether-lumefantrine stock-outs at public health facilities in Kenya. Malar J.

[43] Githinji S., Kigen S., Memusi D. (2013). Reducing stock-outs of life saving malaria commodities using Mobile phone Text-Messaging: SMS for life Study in Kenya. PLoS One.

[44] Malik M., Hassali M.A.A., Shafie A.A., Hussain A. (2013). Why hospital pharmacists have failed to manage antimalarial drugs stock-outs in Pakistan? A qualitative insight. Malaria Research and Treatment.

[45] Mikkelsen-Lopez I., Tediosi F., Abdallah G. (2013). Beyond antimalarial stock-outs: implications of health provider compliance on out-of-pocket expenditure during care-seeking for fever in South East Tanzania. BMC Health Serv Res.

[46] Chipwaza B., Mugasa J.P., Mayumana I., Amuri M., Makungu C., Gwakisa P.S. (2014). Self-medication with anti-malarials is a common practice in rural communities of Kilosa district in Tanzania despite the reported decline of malaria. Malar J.

[47] Kabaghe A.N., Phiri M.D., Phiri K.S., Van Vugt M. (2017). Challenges in implementing uncomplicated malaria treatment in children: a health facility survey in rural Malawi. Malar J.

[48] Sirotich E., Kennedy K., Surangiwala S. (2020). Antimalarial drug shortages during the COVID-19 pandemic: results from the Global Rheumatology Alliance Patient Experience Survey [abstract]. Arthritis Rheumatol.

[49] Mengesha H.Y., Gebrehiwot G.M., Workneh B.D., Kahissay M.H. (2021). Practices of anti-malaria pharmaceuticals inventory control system and associated challenges in public health facilities of Oromiya special zone, Amhara region, Ethiopia. BMC Public Health.

[50] Aberese-Ako M., Magnussen P., Ampofo G.D., Gyapong M., Ansah E., Tagbor H. (2021). An ethnographic study of how health system, socio-cultural and individual factors influence uptake of intermittent preventive treatment of malaria in pregnancy with sulfadoxine-pyrimethamine in a Ghanaian context. PLoS One.

[51] Tefera B.B., Tafere C., Yehualaw A. (2022). Availability and stock-out duration of essential medicines in Shegaw Motta general hospital and Motta Health Centre, North West Ethiopia. PLoS One.

[52] Frosch A.E., Thielen B.K., Alpern J.D. (2022). Antimalarial chemoprophylaxis and treatment in the USA: limited access and extreme price variability. J Trav Med.

[53] Capstick T.G.D., Laycock D., Lipman M.C.I., UK Coalition To Stop TB (2011). Treatment interruptions and inconsistent supply of anti-tuberculosis drugs in the United Kingdom. Int J Tubercul Lung Dis.

[54] Seaworth B.J., Field K., Flood J. (2013). Interruptions in supplies of second-line antituberculosis Drugs—United States, 2005-2012. JAMA.

[55] Ballif M., Nhandu V., Wood R. (2014). Detection and management of drug-resistant tuberculosis in HIV-infected patients in lower-income countries. Int J Tubercul Lung Dis.

[56] Singh A.R., Kharate A., Bhat P. (2017). Isoniazid preventive therapy among children living with tuberculosis patients: is it working? A mixed-method Study from bhopal, India. J Trop Pediatr.

[57] Koomen L.E.M., Burger R., Van Doorslaer E.K.A. (2019). Effects and determinants of tuberculosis drug stockouts in South Africa. BMC Health Serv Res.

[58] Sintayehu K., Zeleke E.D., Temesgen B. (2022). Determinants of stock-outs of first line anti-tuberculosis drugs: the case of public health facilities of Addis Ababa city administration health bureau, Addis Ababa, Ethiopia. BMC Health Serv Res.

[59] Otto-Knapp R., Edwards S., Kuchukhidze G. (2024). Availability of drugs for the treatment of multidrug-resistant/rifampicin-resistant tuberculosis in the World Health Organization European Region, October 2023. Euro Surveill.

[60] Nabity S.A., Agraz-Lara R., Bravo A. (2024). *Notes from the field* : supply interruptions of First- and second-line oral drugs to treat tuberculosis during the previous 12 months — california, January–March, 2023. MMWR Morb Mortal Wkly Rep.

[61] Sreevidya PA, Acharya S, Raul MU, Prakash A (2025). Implementation of isoniazid preventive therapy among children living with diagnosed pulmonary tuberculosis patients: a mixed methods study from Mumbai, India. Trop Med Int Health.

[62] Mekonen Z.T., Fenta T.G., Nadeem S.P., Cho D.J. (2024). Global health commodities supply chain in the era of COVID-19 pandemic: challenges, impacts, and prospects: a systematic review. J Multidiscip Healthc.

[63] Resilient and sustainable systems for health. https://www.theglobalfund.org/en/resilient-sustainable-systems-for-health/.

[64] The shift to integrated supply chains in global health. https://www.pamsteele.org/blog-articles/the-shift-to-integrated-supply-chains-in-global-health/.

[65] Quantification and Forecasting Health implementation manual | UNDP. https://healthimplementation.undp.org/functional-areas/health-product-management/quantification-and-forecasting/.

[66] Havlir D., Gandhi M. (2015). Implementation challenges for long-acting antivirals as treatment. Curr Opin HIV AIDS.

[67] Abrams E.J., Capparelli E., Ruel T., Mirochnick M. (2022). Potential of long-acting products to transform the treatment and prevention of Human Immunodeficiency Virus (HIV) in infants, children, and adolescents. Clin Infect Dis.

[68] Gulma K.A. (2023). Exploring the logistics and supply chain services of HIV/AIDS facilities in north-western Nigeria. Explor Res Clin Soc Pharm.

[69] Institute of Medicine (US) (2012). Facing the reality of drug-resistant tuberculosis in India: challenges and Potential Solutions: summary of a joint workshop by the Institute of Medicine, the Indian National Science Academy, and the Indian Council of Medical research.

[70] Frescura L., Godfrey-Faussett P., Feizzadeh A., El-Sadr W., Syarif O., Ghys P.D. (2022). Achieving the 95 95 95 targets for all: a pathway to ending AIDS. PLoS One.

