# Supplementary material for: Drug stock-outs in HIV, tuberculosis, and malaria programs: a systematic review
Source: eClinicalMedicine. 2026 Jul 31;98:104119. doi: 10.1016/j.eclinm.2026.104119 (PMC13452151; doi:10.1016/j.eclinm.2026.104119)
Supplement: Supplementary file 1 [file mmc1.docx]

**Supplementary Material: Drug Stock‐Outs in Human Immunodeficiency Virus (HIV), Tuberculosis, and Malaria Programs: A Systematic Review**

**Authors:** Avaneesh Kumar Pandey^1^, Ritika Kondel Bhandari^1^, Esmita Charani^2,3^, Jennifer Cohn^4^, Amrita Ghautare^5^, Anne-Grete Märtson ^6^, Uttara Gadde^7^, Sipho Dlamini^2^, Malcolm Miller^2^, Samir Malhotra^1^, Thomas Tängdén^8^, Sanjeev Singh^9^, Marc Mendelson^2^, Nusrat Shafiq^1*^

**Table of contents**

| **Item** | **Page** |
| --- | --- |
| Table S1. Final search terms for the databases | 2 |
| Figure S1. Risk of bias assessment of included studies using the Joanna Briggs Institute (JBI) Critical Appraisal Tool | 3 |
| Table S2. Summary of included evidence in the systematic review of drug stock-outs in Human Immunodeficiency Virus (HIV), Tuberculosis, and Malaria Programs | 4-44 |
| Table S3. Distribution of studies reporting stock-outs by disease category, geographic region, country, and income classification | 45-47 |

(((((((((((((((((((((((((((((((((((((((((((((((((((((((((((((((((((((((((((Antitubercular drugs) OR (Antiretroviral drugs)) OR (Linezolid)) OR (Levofloxacin)) OR (Moxifloxacin)) OR (Clarithromycin)) OR (Thioacetazone)) OR (Ethionamide)) OR (Cycloserine)) OR (Kanamycin)) OR (Capreomycin)) OR (Amikacin)) OR (rifapentine)) OR (Rifabutin)) OR (Streptomycin)) OR (Streptomycin)) OR (Pyrazinamide)) OR (Rifampicin)) OR (Isoniazid)) OR (Chloroquine)) OR (mepacrine)) OR (Quinacrine)) OR (quinine)) OR (mefloquine)) OR (halofantrine)) OR (artemisinins)) OR (artemisinin)) OR (atovaquone)) OR (Proguanil)) OR (dihydroartemisinin 2-(biphenyl-2-yloxy)ethyl ether)) OR (dihydroartemisinin)) OR (artesunate)) OR (artemether)) OR (arteether)) OR (Halofantrine)) OR (Lumefantrine)) OR (HIV Fusion Inhibitors)) OR (ENTRY inhibitors)) OR (Ibalizumab)) OR (Enfuvirtide)) OR (Maraviroc)) OR (HIV Integrase Inhibitors)) OR (Integrase Inhibitors)) OR (Raltegravir)) OR (Raltegravir Potassium)) OR (elvitegravir)) OR (dolutegravir)) OR (bictegravir)) OR (Cobicistat)) OR (Indinavir)) OR (Ritonavir)) OR (Nelfinavir)) OR (amprenavir)) OR (Tipranavir)) OR (Atazanavir)) OR (Atazanavir Sulfate)) OR (Reverse Transcriptase Inhibitors)) OR (nucleoside reverse transcriptase inhibitors)) OR (nonnucleoside/non-nucleotide reverse transcriptase inhibitors)) OR (Zidovudine)) OR (Didanosine)) OR (Stavudine)) OR (Lamivudine)) OR (Emtricitabine)) OR (Zalcitabine)) OR (abacavir)) OR (Tenofovir)) OR (efavirenz)) OR (Nevirapine)) OR (etravirine)) OR (Delavirdine)) OR (Rilpivirine)) OR (doravirine)) OR (Protease Inhibitors)) OR (HIV Protease Inhibitors)) AND (((((Shortages) OR (Shortfall)) OR (Unavailability)) OR (Shock out)) OR (Withdrawn))

**Table S1- Final search terms for the databases**

**Fig S1. Risk of bias assessment of included studies:** Summary of methodological quality of included studies assessed using the Joanna Briggs Institute (JBI) Critical Appraisal Tool. Studies are categorised as having low, moderate, or high risk of bias based on predefined criteria.

| **Study/Year/Country** | **Title** | **Study Design** | **Aims and Objectives** | **Population Involved** | **Gender (Male/Female)** | **Sample Size** | **List of Drugs in Shortage** | **Causes for Shortages** | **Management of Shortages** | **Suggested Mitigation** | **Clinical Impact** | **Economic Impact** |  |
| --- | --- | --- | --- | --- | --- | --- | --- | --- | --- | --- | --- | --- | --- |
|  | **Studies reporting unavailability of anti-HIV drugs** | | | | | | | | | | | | |
| Carmody et al 2003; Brazil^66^ | An evaluation of antiretroviral HIV/AIDS treatment in a Rio de Janeiro public clinic | Retrospective Cross-sectional study with a convenience sample. | To evaluate medication adherence levels among patients on antiretroviral therapy (ART) and explore factors influencing adherence, including psychosocial and healthcare-related factors. | Adults living with HIV/Acquired Immunodeficiency Syndrome (AIDS) in Rio de Janeiro receiving ART through public healthcare, specifically from teaching hospitals and NGOs. | 37/22 | 59 participants | Specific drugs in shortage or stockout were not explicitly identified in the article. | Not detailed in the study; the focus was primarily on adherence and psychosocial factors rather than supply chain issues. | Not directly addressed in the study. | Strengthening adherence support through patient education; psychosocial interventions; and improved communication between healthcare providers and patients. | Non-adherence to ART | Not mentioned |  |
| Marcellin et al 2008; Cameroon^67^ | Determinants of unplanned antiretroviral treatment interruptions among people living with HIV in Yaoundé, Cameroon (EVAL survey, ANRS 12-116) | This was an observational, cross-sectional study conducted as part of the EVAL survey (ANRS 12-116). Data were collected from people living with HIV/AIDS (PLWHA) who were undergoing ART. | The primary aim was to identify the correlates of unplanned interruptions in ART among PLWHA. The study sought to investigate how various socio-demographic, behavioural, and healthcare system factors (e.g., drug availability, social habits) contributed to non-adherence to ART in resource-limited settings like Cameroon. | The study focused on 533 HIV-positive individuals undergoing ART treatment in Yaoundé, Cameroon, and its surrounding neighbourhoods. Participants were recruited from healthcare facilities in this region, reflecting diverse socio-economic and healthcare access levels. | 155/378 | The sample comprised 533 individuals, of whom 68 (12.8%) reported ART interruptions longer than two consecutive days during the previous four weeks. Sixty-four patients (12.0%) reported having interrupted ART for more than 1 month, at least once since ART initiation. This sample allowed for robust statistical analyses of factors associated with treatment interruptions. | Specific antiretroviral drugs were not explicitly detailed in the sources, but the study broadly highlighted stockouts of ART medications as a significant contributor to treatment interruptions. Commonly used ART regimens in resource-limited settings like Cameroon typically include drugs such as zidovudine, lamivudine, efavirenz, nevirapine, and stavudine. | The study identified pharmacy stock shortages as a primary factor linked to ART interruptions. This was attributed to inadequate resource allocation, poor supply chain management, and logistical challenges in delivering ART in a timely and consistent manner. | Not mentioned | Not Mentioned | Not mentioned | Not mentioned |  |
| Bernays et al 2009; Serbia and Montenegro^18^ | Experiencing uncertain HIV treatment delivery in a transitional setting: qualitative study | Qualitative study using in-depth interviews analyzed thematically to explore experiences of HIV treatment access in a transitional setting (Serbia and Montenegro). | To understand the experiences and challenges faced by people living with HIV (PLHIV) and service providers in navigating uncertain HIV treatment delivery. | 41 PLHIV- 18 HIV treatment service providers in Serbia and Montenegro | Not reported | 59 participants | Specific drugs were not listed, but the focus was on antiretroviral therapy (ART) necessary for managing HIV. | Weak supply chains | Treatment interruptions until supplies became available; Changing or rationing treatment regimens; Informal exchanges of medication among PLHIV; Deliberate withholding of information to reduce anxiety among patients. | Not Mentioned | Increased patient anxiety and psychological distress; Reduced trust in the healthcare system and the efficacy of HIV treatment; Uncertainty in managing HIV as a chronic illness; Interruption of antiretroviral therapy potentially impacting treatment outcomes | Not mentioned |  |
| Henery Wasswa 2009, Uganda^13^ | Uganda runs out of antiretroviral drugs after shortfall in aid | New article in BMJ | To address the ongoing shortage of ARV drugs in Uganda and its impact on healthcare delivery, particularly for people living with HIV. | Approximately 1.5 million Ugandans living with HIV, with 820,000 currently on ARV treatment. | Not reported | Not specified issue impacts patients nationwide. | ARV medications and Septrin (cotrimoxazole) | Funding gaps due to delayed aid and mismanagement. | The Global Fund provided an advance supply of ARV drugs, ensuring short-term availability. | Not Mentioned | Increased risk of opportunistic infections,17 patients died due to unavailability of the anti-HIV drugs. | Not mentioned |  |
| Muhamadi et al 2010; Uganda^21^ | Inadequate pre-antiretroviral care, stock-out of antiretroviral drugs and stigma: Policy challenges/bottlenecks to the new WHO recommendations for earlier initiation of antiretroviral therapy (CD<350 cells/ µL) in eastern Uganda | A qualitative study combining methods: 1. In-depth interviews: Conducted with healthcare workers, policymakers, and patients. 2. Focus group discussions (FGDs): Conducted among community members and HIV support groups. | To evaluate challenges in implementing WHO guidelines for earlier ART initiation (CD<350 cells/µL) in resource-constrained settings. | Primary population: People living with HIV/AIDS (PLHIV) in rural and peri-urban areas of Eastern Uganda. - Healthcare workers: Doctors, nurses, clinical officers, and pharmacists in HIV care. - Policymakers and stakeholders: District health officials and program managers. - Affected population: HIV patients experiencing treatment interruptions, including vulnerable groups like pregnant women and children on ART. | 9/11 | Participants for interviews (20) and FGDs (112) | ARVs | Lack of global fund | Not mentioned | Not Mentioned | On patients: 1. Interruptions in ART increased the risk of viral rebound and opportunistic infections. 2. Poor adherence led to higher chances of developing drug-resistant HIV strains. 3. Delay in initiating therapy resulted in advanced disease at presentation. - On healthcare systems: 1. Increased burden of managing complications from treatment interruptions. 2. Higher demand for hospital admissions and second-line therapies. | Not mentioned |  |
| Pasquet et al 2010, Coˆted’Ivoire^11^ | Impact of Drug Stock-Outs on Death and Retention to Care among HIV-Infected Patients on Combination Antiretroviral Therapy in Abidjan, Coˆted’Ivoire | Descriptive observational study conducted from February 2006 to February 2008 in three HIV care centres in Abidjan, Coˆted’Ivoire. | To evaluate the impact of drug stock-outs on HIV treatment outcomes, including mortality, retention to care, and the need for treatment changes. | HIV-infected adults on combination antiretroviral therapy (cART) at three HIV care centres. The focus was on those affected by stock-outs. | 415/1139 | 1,554 study subjects | Nevirapine (NVP), Zidovudine/lamivudine (ZDV-3TC), and other ARVs used in first-line treatment. | Supply chain failures, procurement issues, logistical delays, financial constraints, and bureaucratic inefficiencies. | Regimen modifications including switching to alternative ARVs. Some patients faced delayed treatment and interruptions, which could cause complications. | Improved forecasting, better supply chain management, coordination between stakeholders, and increased funding for continuous drug availability. | Drug stock-outs led to 72 prolonged treatment discontinuations and 98 regimen modifications, representing 11% of the 1,554 patients who started cART. These stock-outs were responsible for 9% of all cART discontinuations and 30% of all cART regimen modifications. High rate of deaths reported in the discontinuations group. | Not mentioned |  |
| Sued et al 2011; Latin America^7^ | HIV drug and supply stock-outs in Latin America | A survey conducted by the Pan American Health Organization (PAHO) across 12 countries in Latin America. | To characterize stock-outs or episodes at risk of stock-outs affecting antiretroviral (HIV) supplies in Latin America. | HIV patients and public health services relying on antiretroviral treatment in 12 Latin American countries. | Not reported | Data was collected on 90 stock-out events and 89 risk-of-stock-out episodes. | Zidovudine + Lamivudine, Abacavir + Lamivudine, Abacavir, Lamivudine, Saquinavir, Tenofovir, Lopinavir, Raltegravir, Fosamprenavir Calcium. | Delays in bidding process (29%); Delays in acquisition (13%); Distribution problems (10%); Drug production difficulties (9%). | Emergency purchases (46%); Changing providers (15%); Changing regimens (11%). | Establish efficient procurement and supply mechanisms for antiretrovirals integrated into health systems, supported by new regional policies and international collaboration. | Unplanned treatment interruptions leading to: - Increased risk of HIV drug resistance; Treatment failure; Discontinuation of laboratory services in 88% of episodes. | Increased costs due to emergency purchases and inefficiencies in the supply chain. |  |
| Pierre-Marie David 2014^22^ | Towards the embodiment of biosocial resistance? How to account for the unexpected effects of antiretroviral scale-up in the Central African Republic | Observant participation, ethnographic interviews, and focus groups conducted across health centres in CAR between 2010 and 2011. | To explore the biopolitical consequences of ARV scale-up and the unexpected effects of drug shortages on health systems and patients in CAR. | Health personnel, institutional representatives, and patients in public and NGO-run AIDS clinics across CAR, particularly in Bangui. | Not reported | Data were gathered through participation in three clinics (three days per week for three months), interviews, and focus groups with community associations, though specific sample sizes are not quantified. | First-line ARVs including nucleoside inhibitors (AZT/3TC, d4T/3TC) and non-nucleoside inhibitors (nevirapine, efavirenz). |  |  | Not Mentioned | Interrupted treatment regimens led to health deterioration and reduced trust in health systems among patients. | Not mentioned |  |
| Fokam et al 2015; Cameroon^31^ | Monitoring HIV Drug Resistance Early Warning Indicators in Cameroon: A Study Following the Revised World Health Organization Recommendations | The study employed a retrospective analysis of HIV Drug Resistance Early Warning Indicators (EWIs) based on data collected between October 2012 and September 2013. The data was gathered from 15 ART sites located in both urban and rural regions of Cameroon, encompassing primary, secondary, and tertiary healthcare levels. It followed the revised 2012 WHO guidelines for monitoring EWIs, ensuring standardized evaluation across diverse healthcare settings. | The primary aim was to assess HIVDR EWIs to support the sustainability of ART programs and prevent the emergence of drug resistance. Specific objectives included: (1) Evaluating ART dispensing practices, (2) Measuring patient retention and adherence, (3) Identifying the incidence of pharmacy drug stockouts, and (4) Proposing interventions to minimize risks of HIVDR. The focus was on optimizing treatment performance and addressing systemic inefficiencies. | The study evaluated patients on ART across 15 sites. This included: - 10 urban and 5 rural ART sites. - Sites varied by healthcare level: primary (7), secondary (3), and tertiary (5). - These patients represented a mix of demographic groups in resource-limited settings, including children, adults, and pregnant women. The settings reflect the diversity in HIV burden and healthcare access within Cameroon. | Not reported | The survey included data from 15 ART sites. Specific patient numbers were not aggregated across sites but were sufficient to evaluate EWIs like pharmacy refill compliance, patient retention, and stock continuity. For example, retention data were derived from monitoring first-year ART patients to assess adherence trends. | Antiretrovirals (ARVs) commonly prescribed in Cameroon, such as first-line and second-line ART regimens, were implicated in pharmacy stockouts. Specific drugs were not named in the study but included those essential for sustaining ART adherence, based on WHO-recommended regimens in resource-limited settings. | The study identified systemic and patient-related causes for drug shortages, including: - Inefficient drug supply chains and logistical challenges in rural areas; Poor planning and procurement mechanisms; Delays in pill pick-up by patients, particularly in rural settings, where adherence rates were lower. - Limited infrastructure and human resources in primary healthcare centres. | Not mentioned | The study proposed: - Strengthening national drug supply systems for better forecasting and distribution. | Stockouts and delayed ART refills can disrupt treatment adherence, leading to the emergence of HIVDR. This compromises the efficacy of first-line treatments and escalates the need for costlier second-line therapies, affecting long-term patient outcomes. Higher rates of resistance were anticipated in rural settings with greater barriers to care access. | Not mentioned |  |
| Nyogea et al 2015; Tanzania^42^ | An assessment of the supply chain management for HIV/AIDS care and treatment in Kilombero and Ulanga districts in southern Tanzania | Exploratory and cross-sectional study conducted in September 2011. | To evaluate the supply chain management of Comprehensive Treatment Centres (CTCs) for HIV/AIDS in Kilombero and Ulanga districts, examine patient management, and assess coping strategies during drug and test kit stock-outs. | Healthcare staff at 12 HIV care sites and HIV/AIDS patients receiving ART in Kilombero and Ulanga districts. | Not reported | 12 healthcare sites surveyed, with structured interviews involving staff and patients. | Antiretrovirals (ARVs), co-trimoxazole, rapid diagnostic test kits for HIV. | Poor transport chain; Delayed in order placement; Unavailability of drug at central distribution centre; No designated personal for procurement. | Substituting ARV regimens based on availability, halting HIV testing, temporary closure of clinics, and patients skipping doses of ARVs or co-trimoxazole due to lack of availability. | Strengthening health management information systems, investing in mobile health (mHealth) solutions, and enhancing human resource capacity. | Not mentioned | There were significant differences in costs incurred between seeking health care in CTCs and CDCI. Visiting a peripheral CTC costed US$ 8.7 less on transport and US$ 5.3 less on accommodation. It took three hours less to reach the CTC than the CDCI. |  |
| Barnett et al 2016; Malawi^48^ | Assessment of non-standard HIV antiretroviral therapy regimens at Lighthouse Trust in Lilongwe, Malawi | Cross-sectional study analyzing all adult patients on non-standard antiretroviral therapy (NS-ART) regimens at Lighthouse Trust, Lilongwe, Malawi, as of February 2012. | To identify factors driving the use of NS-ART regimens and determine the feasibility of transitioning patients back to standard regimens as per Malawi’s national treatment guidelines. | 92 adult HIV-positive patients (mean age 42.4 years; 57% female), receiving NS-ART at the time of study. Majority (91%) on modified second-line NS-ART regimens; others on first-line modified regimens. | 41/52 | 92 confirmed cases (initial review identified 106, but only 92 were verified for NS-ART). | AVRs | Not mentioned | Use of interim NS-ART regimens (e.g., lopinavir/ritonavir monotherapy, alternative NRTI-sparing regimens) during stockout periods. Monitoring and transitioning back to standard regimens once drug supplies were restored. | Implementation of specialized procurement channels for rare or non-standard drugs; Strengthening supply chain forecasting and management; Routine patient medication reviews to avoid prolonged use of NS-ART initiated due to temporary shortages. | Not mentioned | Not discussed |  |
| Poku et al 2017; Ghana^14^ | HIV antiretroviral medication stock-outs in Ghana: contributors and consequences | The study used a qualitative approach with semi-structured interviews. Participants included women living with HIV (WLHIV) and professionals from governmental, non-governmental, and healthcare organizations involved in HIV-related work. The interviews were analyzed using coding and mapping techniques to identify recurrent themes and systemic issues affecting ARV supply. | The primary goal was to understand the causes, management strategies, and impacts (both clinical and economic) of ARV stock-outs in Ghana. It aimed to provide insights into how systemic inefficiencies could be addressed and to propose sustainable solutions to minimize future stock-outs. | The study focused on 40 women living with HIV (WLHIV) who directly experienced ARV shortages and 15 professionals (including policymakers and healthcare workers) involved in HIV-related services. These groups provided firsthand accounts of the challenges and operational bottlenecks within the ARV supply chain. | Professionals= 6/9  WLHIV= 40 | The study interviewed 55 participantscomprising 40 WLHIV and 15 professionals. | Specific antiretroviral drugs are not explicitly named in the study, but the shortages encompassed a range of ARVs critical for HIV treatment. The broad implication is that patients faced interruptions in receiving their prescribed ART regimens. | Key contributors to ARV shortages included: - Inefficient supply chains; Poor inventory management and delays in procurement; Port delays: Inadequate coordination with port authorities led to holdups in clearing imported drugs; Funding gaps: Insufficient government funding for ARV procurement; Over-reliance on foreign aid: Dependence on international donors created vulnerabilities when aid was delayed or reduced. | During shortages, healthcare facilities resorted to redistributing available stocks among themselves, often resulting in uneven access across regions. In some cases, rationing was implemented, forcing patients to modify or delay treatment. These stopgap measures were insufficient and unsustainable, leading to widespread disruptions in therapy continuity. | Recommendations include: - Developing in-country production capacity for ARVs to reduce dependence on imports, ensuring products meet WHO standards; Reviewing and revamping the national drug supply chain to enhance efficiency and minimize bottlenecks; Increasing government investment in ARV procurement to reduce reliance on foreign aid and ensure sustainability. | Disruptions in ART regimens caused inconsistent therapy, potentially leading to increased viral resistance and poorer health outcomes. Patients also faced social stigma due to missed doses, as these lapses were often misconstrued as personal negligence rather than systemic failure. | Stock-outs imposed financial burdens on patients, including the cost of traveling to other facilities to obtain medications and purchasing drugs at private pharmacies when public stocks were unavailable. These expenses were particularly challenging for low-income patients, exacerbating socioeconomic inequities. |  |
| Brennan et al 2017; South Africa^40^ | Tenofovir stock shortages have limited impact on clinic- and patient-level HIV treatment outcomes in public sector clinics in South Africa | A retrospective cohort study examining data from 1 July 2011 to 31 March 2013. It compared patient and clinic outcomes before, during (1 Feb 2012 - 31 Aug 2012), and after a tenofovir stock shortage at four South African clinics. | To assess the effects of tenofovir shortages on ART initiation, adherence, clinic attendance, treatment interruptions, and patient outcomes, including viral load control and loss to follow-up. | HIV-positive individuals attending four public-sector clinics. Two clinics experienced tenofovir shortages; two served as controls. Patients ranged in age, sex, and clinical characteristics (e.g., CD4 counts and comorbidities). | 386/657 | 1043 patients: some initiated on ART, others already undergoing treatment. During the shortage, patients were switched to stavudine or zidovudine based on availability and contraindication. | Tenofovir disoproxil fumarate, the recommended nucleoside reverse transcriptase inhibitor (NRTI) in first-line ART regimens. | Disruptions in the local supply chain affecting two clinics. Shortages were not nationwide but affected about 20% of clinics. Contributing factors likely included logistical and procurement challenges. | Patients were temporarily switched to stavudine or zidovudine. Clinicians followed guidelines to substitute older NRTIs in cases of contraindications and switched back to tenofovir when it became available. | Recommendations include improving procurement systems, ensuring buffer stock availability, and developing early warning systems for stockouts. Training for healthcare workers on handling shortages was also suggested. | No significant impact on ART initiation, adherence, or retention in care. Some patients experienced temporary toxicity from substitute drugs, but viral load suppression rates and loss-to-follow-up remained consistent. Proportion of patients having a missed pharmacy visits increased during shortages. | A decrease in average days between pharmacy visits during  the shortage. |  |
| Meloni et al 2017; Nigeria^26^ | Drug resistance patterns following pharmacy stock shortage in Nigerian Antiretroviral Treatment Program | A retrospective cross-sectional evaluation analyzing plasma samples collected from ART patients between December 2004 and August 2005. The study assessed virologic failure among patients who experienced treatment interruptions compared to those who did not during a national ART drug shortage in late 2003. | To evaluate the impact of unstructured treatment interruptions caused by ART drug shortages on the development of DRMs. The study aimed to quantify the difference in drug resistance profiles between patients who experienced stockouts and those who maintained continuous treatment. | Adults living with HIV enrolled in the Nigerian National ART Program at Jos University Teaching Hospital (JUTH). Participants were divided into two groups: those who experienced treatment interruptions (n=28) and those who did not (n=28). | 34/22 | 56 participants: 28 patients with treatment interruptions and 28 patients with uninterrupted ART. | Key antiretroviral drugs impacted by the shortages included stavudine (d4T) and zidovudine (AZT). | The shortage arose due to: Rapid expansion of ART services without adequate planning for drug procurement and distribution. Weak healthcare infrastructure and supply chain management. Lack of preparedness for scaling up the national ART program. | Not mentioned | Not Mentioned | The study reported significant clinical consequences of drug stockouts: Patients who experienced interruptions had a higher frequency of DRMs (e.g., TAM-2 mutations linked to thymidine analogues like d4T and AZT). Increased resistance to d4T (53.7%) and AZT (64.3%) among interrupted patients. Reduced genotypic sensitivity to second-line ART regimens, limiting treatment options. | Not mentioned |  |
| Moriarty et al 2017; Ghana^34^ | The effect of antiretroviral stock-outs on medication adherence among patients living with HIV in Ghana: A qualitative study | Qualitative study using semi-structured interviews. | The aim of our study was to employ qualitative methods to examine the role of medication stock-outs in contributing to treatment interruption among a sample of patients  already engaged in care for HIV at Komfo Anokye Teaching Hospital in Kumasi, Ghana | Patients living with HIV | 26/31 | 57 patients | Antiretroviral (ARV) drugs (specific names not detailed). | Not mentioned | Changed regimen, stockpiled stock used, Medications from alternative way. | Not Mentioned | 36% reported the drug interruption or stop the treatment due to stockout. | Not mentioned |  |
| Dimala et al 2017; Cameroon^68^ | Motives for change of first-line antiretroviral therapy regimens in an unselected cohort of HIV/AIDS patients at a major referral centre in South-west Cameroon | A retrospective cross-sectional study analysed existing patient data from medical records to identify patterns, causes, and outcomes of changing first-line antiretroviral therapy (ART) regimens. | Focused on identifying the reasons for changes in ART regimens, including patient-specific issues (e.g., side effects), drug-related factors (e.g., toxicity), and systemic issues (e.g., drug shortages). | HIV-positive individuals receiving care at a referral centre in South-West Cameroon, encompassing diverse demographic and clinical backgrounds. | 30/70 | Involved 100 patients, providing a substantial data set to assess trends and causes for changes in ART regimens. | Highlighted issues with commonly used first-line ART drugs, including Tenofovir (TDF), Lamivudine (3TC), and Efavirenz (EFV), which are critical in controlling HIV infection. | Not mentioned | Not mentioned | Not Mentioned | Unplanned treatment interruptions and change in the regimen | Not mentioned |  |
| Bajunirwe et al 2018; Uganda^23^ | Towards 90-90-90 Target: Factors Influencing Availability, Access, and Utilization of HIV Services- A Qualitative Study in 19 Ugandan Districts | Qualitative study using interviews and focus group discussions across 19 districts in Uganda. | To identify barriers to the availability, access, and utilization of HIV services, and suggest strategies to improve service delivery towards 90-90-90 targets. | HIV-positive individuals, healthcare workers, and community stakeholders across 19 Ugandan districts. | 18/14 | 32 multiple stakeholders | Common shortages included antiretroviral drugs (ARVs), cotrimoxazole, and HIV test kits. | Delayed procurement processes, supply chain inefficiencies, inadequate funding, and over-reliance on donor support. | Redistribution of stock between facilities, rationing supplies, and community referrals to better-equipped centres. | Strengthening supply chain systems, increasing domestic funding, and ensuring real-time inventory tracking. | Delayed initiation of treatment or Treatment interruptions led to poor adherence and resistance; stockouts imposed additional costs on patients needing to travel to other facilities. |  |  |
| Tong et al 2018; Cameroon^41^ | Treatment interruption in HIV-positive patients followed up in Cameroon’s antiretroviral treatment programme: individual and health care supply-related factors (ANRS-12288 EVOLCam survey) | Cross-sectional, mixed-methods survey conducted between April and December 2014 in Cameroon. | To document the prevalence of unplanned treatment interruption (TI > 2 days) and identify associated individual and healthcare supply-related factors. | 1885 ART-treated patients across 19 HIV services in Cameroon (Centre and Littoral regions). | 496/1389 | 1885 ART-treated patients. | The study primarily focused on ART stock-outs but did not specify particular drug names. | Not mentioned | Not Mentioned | Not Mentioned | High proportion of patients experiencing treatment interruptions, potentially jeopardizing progress in the fight against HIV. | Not explicitly detailed in the article but inferred that disruptions in treatment could lead to longer-term healthcare costs due to treatment failure. |  |
| Gils et al 2018; Congo^9^ | Stockouts of HIV commodities in public health facilities in Kinshasa: Barriers to end HIV | Cross-sectional survey conducted in April and May 2015 across public health facilities and zonal warehouses in Kinshasa. | To assess the magnitude and duration of stockouts of HIV medicines and diagnostic tests in public health facilities in Kinshasa and evaluate coping strategies during stockouts. | Facilities providing ART to people living with HIV (PLHIV). Targeted facilities with >200 ART patients (high burden) and <200 patients (low burden). | Facility level data | Included 92 facilities (28 high-burden and 64 low-burden) and 27 of 33 zonal warehouses. These facilities collectively served around 22,000 ART patients. | Tenofovir-Lamivudine-Efavirenz (TDF-3TC-EFV); Zidovudine-Lamivudine-Nevirapine (AZT-3TC-NVP); Cotrimoxazole - Determine HIV-1/2 rapid diagnostic tests. | Not mentioned | Substitution with other ART regimens (46% of cases); Patients often not provided with any ART (54% of cases); Referral to other facilities or advice to return later. | Not Mentioned | Not mentioned | Not mentioned |  |
| Hwang et al 2019; South Africa^46^ | Stock-outs of antiretroviral and tuberculosis medicines in South Africa: A national cross sectional survey | National cross-sectional survey conducted via telephone between October and December 2015. | To estimate the prevalence of stock-outs of antiretroviral (ARV) and tuberculosis (TB) medicines in South Africa and understand their clinical and operational impacts. | Survey covered 2370 public health facilities providing ARV and TB treatment across South Africa. Patients relying on these facilities, especially those with HIV and TB, were directly affected. | Facility level data | 2370 health facilities. | Adult ARVs: First-line (11%), second-line (15%), less commonly used (11%)- Pediatric ARVs: 9%- Nevirapine syrup for mother-to-child transmission: 1%- TB medicines: 4%. | Documented national shortage of adult lopinavir/ritonavir; poor supply chain management; uneven stock distribution across provinces. | Borrowing from other facilities- Referral to other facilities- Switching to alternative regimens (often less optimal)- Patient instructions to return later. | Not Mentioned | High impact in 25% of cases: Patients left without treatment or with incomplete regimens; Medium impact in 39%: Suboptimal switches, referrals, or borrowing incomplete supplies; Low impact in 36%: Appropriate alternatives provided. | Not mentioned |  |
| Zakumumpa et al 2019; Uganda^29^ | The number of clients is increasing but the supplies are reducingâ€: provider strategies for responding to chronic antiretroviral (ARV) medicines stock-outs in resource limited settings: a qualitative study from Uganda | Qualitative research design within a larger mixed-methods study. Multiple case-study approach using semi-structured interviews, site visits, and documentary review. | To understand the strategies devised by health facilities in Uganda to respond to chronic stock-outs of ARV medicines, and the operational contexts influencing these strategies. | Health facility staff (ART clinic managers, clinicians, pharmacists) in Uganda, and patients affected by ARV stock-outs. | Not reported | 76 semi-structured interviews conducted with health workers across 16 selected health facilities. | ARV medicines. Specific drugs were not listed in the abstract, but the focus was on ART-related stock-outs. Paediatric formulations. | Issues related to insufficient national-level procurement, poor supply chain management, and inadequate forecasting and stock management practices at the facility level. | Paediatric patients were sent to other facility which have the stock for these drugs. | Not Mentioned | Not mentioned | Not mentioned |  |
| Bernard et al 2022; Kenya^37^ | Impacts of the COVID-19 pandemic on access to HIV and reproductive health care among women living with HIV (WLHIV) in Western Kenya: A mixed methods analysis | Cross-sectional telephone surveys | To assess the impact of the COVID-19 pandemic on access to HIV and family planning services among WLHIV in Kenya. | Women living with HIV (WLHIV) aged 15 years or older, receiving treatment through the AMPATH program in western Kenya. | 0/814 | 814 women were called for surveys; 784 (96%) completed the survey. | Stock-outs reported for HIV medications (mainly ART) and family planning methods (implants, injectables). | Challenges included medication stock-outs, transportation issues, and cost-related difficulties in accessing care. | Women had to adapt by seeking alternative resources, though stock-outs were largely unaddressed during the period. | Improve access to transportation, address medication stock-outs, and enhance distribution systems to ensure consistent supply. | Increased difficulty in obtaining HIV medications and refills, but minimal impact on ART adherence. | Economic strain due to transportation costs and difficulty accessing services, which might exacerbate financial barriers to care. |  |
| Jacobs et al 2024; Kenya and Uganda^32^ | Availability and stock-outs of paediatric antiretroviral treatment formulations a health facility in Kenya and Uganda | Cross-sectional survey | To assess the availability and stock-outs of paediatric ART formulations in health facilities in Kenya and Uganda. | Children living with HIV in Kenya and Uganda, with a focus on treatment access. | Facility level data | 144 health facilities (72 in Kenya, 72 in Uganda), including both public and private-not-for-profit facilities. | Dolutegravir (DTG), abacavir/lamivudine, lopinavir/ritonavir, nevirapine oral solution, and other limited-use ART drugs. | Not mentioned | Delays in supply chain recovery, stockouts lasting 37-60 days, inadequate forecasting and planning. | Not Mentioned | Delays and interruptions in treatment, increased risk of drug resistance, and negative health outcomes for children. | Not mentioned |  |
| Rebecca Irons 2024; Venezuela^44^ | Resistance and the regimen: The microthanatopolitics of Venezuelan antiretroviral scarcity and HIV drug adherence failures | Qualitative study based on interviews and testimonies from HIV activists and Venezuelan migrants living in Colombia. | To explore the impact of ART scarcity on HIV treatment adherence and the development of resistance to ART drugs in Venezuela, both within the country and among migrants. | Individuals living with HIV in Venezuela and Venezuelan migrants in Colombia. | Migrants= 29/11 | 6 interviews with Venezuelan HIV activists (2024), supported by 40 testimonies from Venezuelan migrants living with HIV (2021â€“2024). | Antiretroviral drugs (specific drugs not listed in the summary). | Lack of provision of ART by the Venezuelan state, leading to treatment pauses and adherence failures. The shortages are compounded by political instability and external humanitarian aid. | patient get treatments from NGOs and other alternative ways. | Not Mentioned | Development of drug resistance to ART, reducing future treatment options and potentially accelerating the progression of HIV/AIDS in affected individuals. | Not mentioned |  |
| Sliefert et al 2024; Kenya^52^ | Challenges with paediatric antiretroviral therapy administration: Qualitative perspectives from caregivers and HIV providers in Kenya | Qualitative study with focus groups (FGD) and key informant interviews (KII). | To understand the challenges of paediatric ART administration from the perspectives of caregivers and healthcare providers in Kenya. | Caregivers and healthcare providers in Kenya, particularly those managing paediatric HIV care. Caregivers were mainly mothers, and children ranged from infants to 18 years old. | Caregivers= 12/60  Healthcare providers= 11/19 | 72 caregivers (caring for 83children) and 30 healthcare providers across 3 hospitals in Kenya. | Specific drugs were not mentioned in the study, but the article focuses on the challenges related to ART formulations like syrups and tablets. | Not specifically discussed; instead, the article focuses on formulation-related challenges like poor taste, complex administration, and cold storage. | The focus was on addressing challenges in medication administration, including caregiver education, preparation, and adherence support. | Improve ART formulations, such as reducing bitterness and simplifying administration. Enhanced caregiver and provider training to improve adherence. | Poor adherence due to regimen-related challenges, including child refusal of medication, resulting in potential treatment failure. | Indirect economic impacts, such as increased healthcare costs due to poor adherence and the need for additional interventions to manage non-adherence. |  |
| Loung et al 2025; Australia | Impact of antiretroviral shortages in HIV therapy and prevention uptake in  Australia | Before and after | The aims of this study were to investigate changes in dispensing patterns of tenofovir with emtricitabine  for HIV treatment or PrEP before, during and after the reported shortages and changes in health seeking behaviours | HIV patients on treatment and prevention | Not reported | Not reported | Tenofovir and emtricitabine based formulation | Manufacturing issues as reported by the  Therapeutic Goods Administration | Given different formulation of tenofovir and emtricitabine | Not mentioned | Lead to a reduction in the rate of dispensing of PrEP. Patients have to opt for prescription of an alternative form of PrEP, or go without medication increasing the risk of HIV infection if engaging in sexual activity with HIV-positive people. | Pay an increased price for the medicine to access an overseas-registered product prior to PBS-listing. |  |
| Schmutz et al 2026; Africa, the Asia-Pacific  and Latin America and  the Caribbean. | The implementation of tuberculosis  preventive therapy in HIV care clinics  in Africa, Asia and Latin America: a  multiregional site survey | Survey | Evaluate the implementation of TPT in a global sample of HIV care clinics in LMICs from the International Epidemiology Databases to Evaluate AIDS (IeDEA) consortium in Africa, the Asia-Pacific and Latin America and the Caribbean, with a  focus on TB screening and testing, eligible populations and  TPT regimens available. | HIV patients | Facility level data | 172 HIV clinics | TB preventive regimens (INH based and Rifampicin based) | Not mentioned | Not mentioned | Not mentioned | The HIV patients were not able to start the Tuberculosis prophylaxis | Not mentioned |  |
|  | **Studies reporting unavailability of anti-Malarial drugs** | | | | | | | | | | | | |
| Lufesi et al 2007; Malawi^15^ | Deficient supplies of drugs for life threatening diseases in an African community | Cross-sectional audit and retrospective review conducted in July and August 2005. Health centres were visited to assess drug availability and stock management practices. | To investigate the extent of drug shortages for life-threatening diseases (pneumonia and malaria) in Malawi and identify the underlying causes of these shortages. | Patients seeking treatment for pneumonia and malaria in the Lilongwe District, Malawi. Health centres providing essential drugs for life-threatening conditions. | Not reported | 8 health centres (randomly selected from 37 in the district); 8,968 patient records were reviewed to assess the dispensed quantities of drugs over a one-month period. | 1. Pneumonia drugs: Benzyl penicillin (4% supply), other drugs for pneumonia treatment. 2. Anti-malarial drugs: Quinine injections (9% supply), SP and para-aldehyde. | 1. Insufficient deliveries from the Regional Medical Store (main cause). 2. Logistical failures and discrepancies between Stock Cards and actual stock levels. 3. uneven distribution of drugs. Delay in ordering and supply. | Not mentioned | Not Mentioned | Not mentioned | Not mentioned |  |
| Njogu et al 2008; Kenya^54^ | Health facility and health worker readiness to deliver new national treatment policy for malaria in Kenya | Cross-sectional survey conducted across health facilities in four sentinel districts in Kenya. | To evaluate the readiness of health facilities and health workers to deliver the new artemether-lumefantrine (AL) treatment policy for uncomplicated malaria. | The study involved 211 government health facilities and 654 health workers providing outpatient consultations in four districts. | 281/373 | 211 health facilities and 654 health workers. | Artemether-lumefantrine (AL) tablets (including different weight-specific packs). | Stock-outs were due to supply chain issues, inadequate inventory management, and lack of consistent drug availability. | Not mentioned | Not Mentioned | Not mentioned | Not mentioned |  |
| Kangwana et al 2009; Kenya^12^ | Malaria Drug Shortages in Kenya: A Major Failure to Provide Access to Effective Treatment | Cross-sectional survey | Investigate the availability of artemether-lumefantrine (AL) in government health facilities in Kenya | Health facilities in Kenya, specifically in Nyanza, Western, and Coast Provinces, and the populations needing malaria treatment | Facility level data | 164 health facilities (115 dispensaries, 30 health centres, 19 hospitals) | Artemether-lumefantrine (AL), particularly the weight-specific packs for younger children | Delayed procurement due to confusion over tendering process, international tendering issues, funding issue, lack of quality testing facility | Alternative treatments were used (quinine, amodiaquine, SP), and patients were advised to seek drugs from the private market | Improve procurement processes, ensure timely tendering, avoid delays in drug delivery, and maintain flexibility in procurement channels | Inadequate treatment access, especially for the most vulnerable groups (young children), leading to potential increases in morbidity and mortality from malaria | Delays in drug procurement led to higher costs for emergency procurement and more expensive alternatives used in the interim |  |
| Bogale et al 2015; Ethiopia^57^ | Facilitators and barriers of malaria prevention and treatment services to pregnant women in Ethiopia: a multi-level health system analysis, 2025 | Qualitative study | Aim of the study is to seeks to generate actionable evidence to enhance malaria  programme effectiveness, improve resource utilization,  and ultimately strengthen maternal and neonatal health  outcomes in Ethiopia. | 49 multi-level stakeholders in the prevention and treatment services, including  policymakers, programme managers, advocacy groups, and healthcare service providers | 29/20 | 49 stakeholders | Chloroquine/  primaquine  shortages | Not mentioned | Not mentioned | Not mentioned | Not Mentioned | Not mentioned |  |
| Chuma et al 2010; Kenya^19^ | Research Barriers to prompt and effective malaria treatment among the poorest population in Kenya | A mixed-methods study conducted in four malaria-endemic districts in Kenya. | To identify factors preventing prompt and effective malaria treatment among the poorest populations. The study aimed to provide actionable insights for policies aimed at improving access to artemisinin-based combination therapies (ACTs) in underserved areas. | The study focused on individuals and households in the poorest socio-economic quintile within malaria-endemic regions. This included children under five, who are highly vulnerable to malaria, and their caregivers. | Not reported | 708 households participated in the survey. Discussions and interviews included 24 community focus groups, 34 healthcare providers, and 359 patients exiting health facilities. | Artemisinin-based combination therapies (ACTs), the first-line treatment for malaria. Stockouts were frequently reported in public health facilities, limiting availability. | Key causes included insufficient drug supply to public facilities, funding gaps, inadequate distribution systems, poor inventory management, and seasonal spikes in demand. Transport challenges and unofficial payments for drugs also exacerbated the problem. | Not mentioned | Not mentioned | Not mentioned | Not mentioned |  |
| Alba et al 2010; Tanzania^27^ | Improvements in access to malaria treatment in Tanzania after switch to artemisinin combination therapy and the introduction of accredited drug dispensing outlets- a provider perspective | The study is a longitudinal observational design. - Data collection included: 1. Yearly censuses of drug shops in rural areas. 2. Monthly data on availability of anti-malarial drugs in public health facilities. 3. Retail audits to measure anti-malarial sales volumes across public, mission, and private outlets. 4. Complemented by population-level data from the Demographic Surveillance System (DSS) between 2004 and 2008. | To evaluate the impact of the transition from sulphadoxine-pyrimethamine (SP) to artemether-lumefantrine (ALu) on malaria treatment access in Tanzania. - To assess the role of Accredited Drug Dispensing Outlets (ADDOs) in improving malaria treatment access in the private retail sector. - To measure improvements in drug availability, accessibility, and usage post-policy change. | Rural population in Kilombero and Ulanga districts, and urban population in Ifakara town. - Focus on households within 5 km of drug shops and patients accessing malaria treatment. - Both public and private sector patients were involved, with a special focus on drug shops' reach to rural communities. | Facility level data | 10 public health facilities (health posts, dispensaries) were monitored for anti-malarial drug availability. Multiple private drug outlets and ADDOs were surveyed. - Data were also supplemented by community-level data from the DSS, providing insights from thousands of households. | Sulphadoxine-pyrimethamine (SP): experienced stockouts in health facilities during the switch to ALu (only 40% of months had SP in stock). - Artemether-lumefantrine (ALu): faced stockouts in private drug shops, with the drug found in only 30% of drug shops and no general shops. | SP: Stockouts were primarily due to the transition to ALu. Health facilities were unprepared to manage the switchover, leading to depletion of existing stocks. - ALu: Limited distribution to private drug shops was a major factor. Although ALu was widely available in public health facilities, it was less accessible in rural drug shops, hindering its overall use. | SP: Stockouts of SP were partially managed by private sector availability, where SP was still widely sold in shops despite public health shortages. - ALu: Although there were challenges in retail availability, the stock levels in health facilities remained stable (over 80%). This distribution discrepancy resulted in low utilization of ALu in the broader community, as many could not access it through retail outlets. | Not Mentioned | Not mentioned | Not mentioned |  |
| Barrington et al 2010; Tanzania^51^ | SMS for Life: a pilot project to improve anti-malarial drug supply management in rural Tanzania using standard technology | The study was a 21-week pilot project (2009-2010) conducted across 129 health facilities in three rural districts in Tanzania. It used SMS technology and electronic mapping for drug supply management. | The goal was to improve anti-malarial drug supply chain management by enhancing the visibility of stock levels at health facilities. This aimed to reduce drug stock-outs and ensure timely restocking. | The study involved rural health facilities and the patients they serve. The affected population includes those in areas with high malaria prevalence and poor infrastructure. | Facility level data | The pilot was implemented in 129 health facilities spread across three districts in Tanzania. | Anti-malarial drugs, specifically artemisinin-based combination therapies (ACTs), were reported to be in stock-out or shortage. The study aimed to track these shortages. | Not mentioned | The use of SMS technology helped facilitate weekly stock reporting from health facilities to district managers. This system allowed for quick detection of stock-outs and improved the coordination of restocking. | The project recommended the use of SMS technology for real-time reporting of stock levels and improved communication between health facilities and central management to avoid stock-outs and optimize supply chain management. | Not mentioned | Not mentioned |  |
| Windisch et al 2011; Uganda^17^ | Scaling up antiretroviral therapy in Uganda: using supply chain management to appraise health systems strengthening | Qualitative and observational study. Utilized WHO health systems framework to analyze health systems and supply chain management. | To examine the relationship between antiretroviral therapy (ART) supply chain management and the overall health system in Uganda. | People living with HIV/AIDS in Uganda, healthcare workers, and health system actors involved in ART distribution. | District level data | Data from key informant interviews and health service delivery observations in a study district (specific sample size not mentioned) | ART drugs, including antiretrovirals, in short supply due to supply chain inefficiencies. | Lack of data for forecasting for the requirement, Miss management and poor accountability, Lack of govt funding and dependency on donor funds, | Lending and borrowing the medicines between the facilities, Duo therapy, late initiation for new patients and treatment interruption. | Strengthening supply chain management with a systems perspective, addressing root causes in governance, financing, human resources, and service delivery. | ART treatment interruptions due to stockouts likely resulted in poor health outcomes for people living with HIV. | Shortages increase healthcare costs, reduce the efficiency of ART programs, and impact the sustainability of the health system. |  |
| Sudoi et al 2012, Kenya^55^ | The magnitude and trend of artemether lumefantrine stock-outs at public health facilities in Kenya | The study conducted 18 cross-sectional surveys monthly from January 2010 to June 2011. These surveys included nationally representative samples of public health facilities. Stock-outs were measured for all four weight-specific AL packs: 6, 12, 18, and 24 tablets. | Quantify the magnitude of stock-outs of artemether-lumefantrine (AL) in public health facilities. - Analyze trends in stock-outs to guide policy and interventions. - Provide a baseline for evaluating anti-malarial drug distribution systems. | Malaria patients | Facility level data | A total of 162-176 facilities were surveyed monthly, with variations in the number based on availability and operational challenges during the survey period. | Artemether-lumefantrine (AL) in weight-specific packs: 6, 12, 18, and 24 tablets. - AL is the first-line treatment for uncomplicated malaria under Kenyan guidelines. | Not mentioned | Not mentioned | Not Mentioned | Not mentioned | Not mentioned |  |
| Githinji et al 2013; Kenya^50^ | Reducing Stock-Outs of Life Saving Malaria Commodities Using Mobile Phone Text-Messaging: SMS for Life Study in Kenya | A cluster-randomized, interventional study to reduce stock-outs of malaria commodities using SMS data transmission for stock visibility. | To assess if SMS-based visibility of stock levels could reduce stock-outs of Artemether-Lumefantrine (AL) and rapid diagnostic tests (RDTs) in public health facilities. | Health workers and facilities across five rural districts in Kenya (Machakos, Msambweni, Ijara, Manga, Vihiga). The affected population was those in malaria-endemic areas requiring malaria treatment and diagnostics. | Facility level data | 87 public health facilities across five Kenyan districts, with health workers reporting stock levels. | Artemether-Lumefantrine (AL) in various formulations (6, 12, 18, 24), and Rapid Diagnostic Tests (RDTs). | Not mentioned | The SMS system allowed real-time visibility of stock levels, alerting District Health Management Teams (DHMT) to redistribute stocks from facilities with excess to those with shortages. | Not Mentioned | The reduction of stock-outs of malaria commodities improved access to timely treatment for malaria, which is essential for reducing mortality in endemic regions. | Not mentioned |  |
| Malik et al 2013; Pakistan^24^ | Why Hospital Pharmacists Have Failed to Manage Antimalarial Drugs Stock-Outs in Pakistan? A Qualitative Insight | Qualitative study using semi-structured interviews. | To explore the perceptions of hospital pharmacists regarding the management of antimalarial drugs and the reasons for stock-outs in Pakistan's healthcare system. | Hospital pharmacists working in tertiary healthcare facilities in Islamabad and Rawalpindi, Pakistan. | 11/5 | 16 hospital pharmacists | Antimalarial drugs (specific drugs not mentioned in the study, but antimalarials in general). | Lack of implementation of essential drug list, Unethical promotion of drugs by pharmaceutical industry, Financial constraints, Prescribing practices, Inappropriate calculation of lead time, Lack of generic prescribing | Pharmacists suggested that non-proprietary prescribing, better training for healthcare professionals, implementation of essential drug lists, and adherence to standard treatment guidelines would mitigate stock-outs. | Improved drug management practices, more awareness about standard treatment guidelines, training of health professionals, and a more defined role for pharmacists in drug management. | Irrational treatment of malaria due to stock-outs and mismanagement, potentially leading to worsened patient outcomes. | Not mentioned |  |
| Mikkelsen-Lopez et al 2013; Tanzania^39^ | Beyond antimalarial stock-outs: implications of health provider compliance on out-of-pocket expenditure during care-seeking for fever in South East Tanzania | Retrospective analysis combining ACT stock data (2009â€“2011) from six health facilities and household data from 333 respondents on the cost of fever care-seeking. | To examine how stock-outs of Artemisinin-based Combination Therapy (ACT) and non-compliant health worker behavior affect out-of-pocket expenditures for fever care in Ulanga District, Tanzania. | 333 households in Ulanga District, Tanzania. The population involved includes caregivers seeking treatment for fever. | Not reported | 333 respondents from Ulanga District. | Artemisinin-based Combination Therapy (ACT). | Not mentioned | Some patients sought treatment from private sector providers, but this led to higher out-of-pocket costs. | Not Mentioned | Stock-outs of ACT and non-compliant health workers led to delays in effective malaria treatment, potentially worsening health outcomes. | Increased out-of-pocket expenditures for households, particularly for those who sought care in the private sector due to public sector stock-outs and non-compliance. |  |
| Chipwaza et al 2014; Tanzania^47^ | Self-medication with anti-malarials is a common practice in rural communities of Kilosa district in Tanzania despite the reported decline of malaria | Qualitative study using 12 Focus Group Discussions (FGDs) with community members and 14 in-depth interviews (IDIs) with health workers. | To assess the prevalence of self-medication with anti-malarials in rural communities and identify the reasons behind this practice. | Rural community members in Kilosa district, Tanzania. The study involved 93 participants (56 women, 37 men). Health workers were also interviewed. | FGD= 37/56  Interviews= 6/8 | 93 community members participated in FGDs, and 14 health workers were interviewed. | Anti-malarial | inadequate supply from central pharmacy (Govt) | Not mentioned | Not Mentioned | Risks of misdiagnosis, incorrect drug usage, delayed treatment, and the potential for antimicrobial resistance. | Self-medication could potentially lower healthcare costs for individuals but could also increase long-term economic burden due to poor health outcomes. |  |
| Mikkelsen-Lopez et al 2014; Tanzania^69^ | The challenge to avoid anti-malarial medicine stock-outs in an era of funding partners: the case of Tanzania | Descriptive observational study using SMS technology for weekly reporting on stock availability of artemisinin-based combination therapy (ACT) from over 5,000 health facilities. | To determine the pattern of availability of ACT and to explore the causes of observed stock-outs in public health facilities in Tanzania from mid-2011 to 2012. | Public health facilities in Tanzania, serving the general population, including rural areas and hard-to-reach communities. | Facility level data | Data collected from over 5,000 public health facilities across Tanzania. | Artemisinin-based combination therapy (ACT), specifically artemether-lumefantrine, used for treating malaria. | 1) Insufficient ACT supplies entering Tanzania. 2) Irregularity in the pattern of ACT supply. 3) Bureaucratic delays both within the country and from donors. | Utilized SMS for Life mobile phone reporting tool to track ACT availability across the country, helping improve the transparency and efficiency of the supply chain. | Not Mentioned | Not mentioned | Not mentioned |  |
| Kabaghe et al 2017; Malawi^16^ | Challenges in implementing uncomplicated malaria treatment in children: a health facility survey in rural Malawi | Cross-sectional survey conducted in six rural health centres around Majete Wildlife Reserve in Chikwawa district, Malawi. Data collection methods included interviews with healthcare providers and exit interviews with guardians of children receiving malaria treatment. | - Assess effectiveness of malaria treatment delivery for children under 5. - Identify challenges in ACT prescription, dosing, and availability. - Evaluate the quality of implementation of uncomplicated malaria treatment in rural settings. | - Guardians and children under 5 years presenting with symptoms suggestive of malaria. - Healthcare workers responsible for malaria diagnosis and treatment. | Not reported | 120 children and their guardians were included in the study. Healthcare providers from six health centres contributed to data on service delivery practices and challenges. | - Artemisinin-lumefantrine (first-line ACT for malaria). - Other antimalarial drugs potentially impacted due to inconsistent supply. | Late and inaccurate reporting of the stock leading to delayed restocking. - Drug wastage and over prescription | Not mentioned | #NAME? | Not mentioned | Not mentioned |  |
| Sirotich et al 2020; Global^33^ | Antimalarial Drug Shortages During the COVID-19 Pandemic: Results from the Global Rheumatology Alliance Patient Experience Survey | Online survey distributed via patient support organizations and social media, collecting data on patient demographics, medications, COVID-19 status, and health outcomes. | To assess the impact of antimalarial drug shortages during the COVID-19 pandemic on patients with rheumatic diseases. | Patients with rheumatic diseases, including those in Africa, Southeast Asia, Americas, and Europe, who were prescribed antimalarials for their condition. | 908/8453  Not disclosed= 32 | 9,393 respondents, of which 3,872 (41.2%) were taking antimalarials. | Hydroxychloroquine, chloroquine. | Increased demand during the COVID-19 pandemic, especially as antimalarials were repurposed for COVID-19 treatment despite lacking strong evidence of efficacy. | Not Mentioned | Not Mentioned | Patients who couldn't access antimalarials reported higher disease activity and poorer mental and physical health compared to those who could access their medications. | Not mentioned |  |
| Mengesha et al 2021; Ethiopia^30^ | Practices of anti-malaria pharmaceuticals inventory control system and associated challenges in public health facilities of Oromiya special zone, Amhara region, Ethiopia | Facility-based cross-sectional study using quantitative and qualitative methods (explanatory sequential mixed methods). | To assess anti-malaria pharmaceuticals inventory control practices and associated challenges in public health facilities of Oromiya Special Zone, Amhara Region, Ethiopia. | All public health facilities in Oromiya Special Zone, including 27 health centres and 2 hospitals; key informants: 12 health professionals managing anti-malaria pharmaceuticals. | Facility level survey | Quantitative: 29 facilities. Qualitative: 12 key informants. | Artemether-lumefantrine, artesunate injection, primaquine tablets, chloroquine syrup, rapid diagnostic test (RDT). | - Lack of integrated logistics training. Poor management support. Supply of near-expiry and inadequate quantities from the pharmaceuticals supply agency. Non-consideration of Look-Ahead Seasonality Indices (LSI). | Redistribution of overstocked items in 9 of 29 facilities; emergency orders placed in 13.8% of cases. | Not mentioned | Not mentioned | - Wastage rate of anti-malaria drugs at 11.32% (above national standards).- Overstock leading to storage space issues and inefficiency in resource allocation. |  |
| Aberese-Ako et al 2021; Ghana^45^ | An ethnographic study of how health system, socio-cultural and individual factors influence uptake of intermittent preventive treatment of malaria in pregnancy with sulfadoxine pyrimethamine in a Ghanaian context | The study used an ethnographic approach, combining non-participant observation with case studies, informal conversations, and in-depth interviews (IDIs). | The primary aim was to understand the factors influencing the uptake of intermittent preventive treatment (IPTp) for malaria during pregnancy with sulfadoxine-pyrimethamine (SP). | The study focused on pregnant women receiving ANC in the Ashanti and Volta regions of Ghana, healthcare providers, and community members. | Facility level data | The study involved data collection from 8 health facilities and 8 communities. | Sulfadoxine-pyrimethamine (SP) was identified as the key drug experiencing stockouts or shortages during the study. SP is the recommended drug for intermittent preventive treatment of malaria in pregnancy, and its unavailability directly impacted the delivery of malaria prevention strategies in ANC services. | Not mentioned | In response to drug stockout, sometimes limiting the number of doses administered to pregnant women or charging them for medications that were supposed to be provided free of charge. Drug borrowing from sister facility. | Not Mentioned | Not mentioned | Not mentioned |  |
| Tefera et al 2022; Ethiopia^56^ | Availability and stock-out duration of essential medicines in Shegaw Motta general hospital and Motta Health Centre, North West Ethiopia | Cross-sectional descriptive study | To assess the availability and stock-out duration of essential medicines in Shegaw Motta General Hospital and Motta Health Centre | Patients receiving care at Shegaw Motta General Hospital and Motta Health Centre | Facility level data | The study included all the available essential medicines for a duration of 6 months at the two health facilities. | ART, Antimalarial and anti TB | Not mentioned | Not mentioned | Not Mentioned | Not mentioned | Not mentioned |  |
| Frosch et al 2022; USA^49^ | Antimalarial chemoprophylaxis and treatment in the USA: limited access and extreme price variability | Cross-sectional survey conducted in Minneapolis/Saint Paul and New York City, USA, to assess the availability and cost of antimalarials for chemoprophylaxis and treatment. | To evaluate the barriers to malaria chemoprophylaxis and treatment in the USA, focusing on the availability and cost of antimalarial drugs at retail pharmacies. | Individuals traveling from the USA to malaria-endemic regions, particularly immigrants, international travellers, and tourists. | Facility level data | Data from 21 retail pharmacies in Minneapolis/Saint Paul and New York City, surveyed between 2018 and 2020. | Atovaquone-proguanil, Artemether-lumefantrine, chloroquine, mefloquine, and doxycycline. | Price variability and lack of insurance coverage for antimalarial drugs. Limited access to certain antimalarials due to inconsistent supply chains. High cost of prescription antimalarials compared to over-the-counter alternatives. | Use of alternative drug options (e.g., doxycycline instead of mefloquine).- Referral to pharmacies with better stock availability. | Improving access through expanded insurance coverage for antimalarial medications. Ensuring more consistent supply chains to avoid stockouts. | Not mentioned | High inter-pharmacy variability in pricing, with prices varying more than 10-fold between pharmacies for atovaquone proguanil, doxycycline and chloroquine (30-day trip ranged between $33.90 and $877.50 for atovaquone-proguanil; for doxycycline,between$30.00and$1470.00). |  |
|  | **Studies reporting unavailability of anti-TB drugs** | | | | | | | | | | | | |
| Capstick et al 2011; UK^25^ | Treatment interruptions and inconsistent supply of anti-tuberculosis drugs in the United Kingdom | Cross-sectional survey using questionnaires sent to pharmacists at 168 National Health Service (NHS) TB treatment centres across the UK, focusing on drug supply challenges from 2007 to 2009. | To assess difficulties in obtaining anti-tuberculosis drugs, the resulting impact on treatment regimens, availability of paediatric formulations, and resources available for managing shortages. | TB patients treated at NHS centres, including children requiring liquid formulations and individuals with multidrug-resistant TB (MDR-TB). | Facility level data | 77 treatment centres responded to the survey (46% response rate). | First-line TB drugs for drug-susceptible TB- Second-line drugs for MDR-TB- Licensed liquid formulations for paediatric patients. | Limited availability of specific formulations, especially for paediatric use.- Supply chain inefficiencies and inconsistent procurement strategies.- Lack of national guidance for maintaining adequate drug supplies. | Paediatric centres often resorted to unlicensed, locally prepared liquid formulations with variable strengths.- Treatment interruptions (27% of centres) and regimen alterations (19% of centres) for drug-susceptible TB.- Similar issues reported by 36% of MDR-TB centres, with 16% experiencing interruptions and 5% altering regimens. | certain hospitals could increase stock-holding levels and act as a resource for other local hospitals to source rarely-used drugs such as clofazimine and cycloserine. | 21 (27%) of the 77 Trusts had to interrupt the prescribed treatment course at least once due to drug shortages. Of the 55 Trusts who had treated MDR-TB, therapy was interrupted at least once in nine (16%). Lack of paediatric liquid formulation. | Not mentioned |  |
| Seaworth et al 2013; USA^20^ | Interruptions in Supplies of Second-Line Antituberculosis Drugs â€” United States, 2005â€“2012 | A retrospective analysis of second-line drug (SLD) shortages for tuberculosis (TB) treatment in the U.S. using data from surveys, FDA, CDC, and National Tuberculosis Controllers Association (NTCA) reports between 2005â€“2012. | To assess the frequency, causes, and impact of SLD shortages on TB treatment and to propose strategies to mitigate and prevent these shortages. | TB patients, including those with multidrug-resistant (MDR) and extensively drug-resistant (XDR) TB, across the U.S. Health departments, TB control programs, and healthcare providers were also significantly impacted. | Not reported | Survey data covered approximately 81% of responding jurisdictions that reported MDR TB cases during the study period. Specific patient numbers were not provided. | Capreomycin, cycloserine, ethionamide, kanamycin, amikacin, and para-aminosalicylic acid. These are crucial SLDs for treating MDR and XDR TB. | Nationwide shortages and supply chain issues.- Shipping delays (71% of cases).- Complicated procurement processes for investigational drugs. Lack of early notification to FDA. | Not mentioned | Early notification to FDA about potential shortages.- Creation of a centralized stockpile for critical TB drugs.- Collaboration between CDC, FDA, pharmaceutical companies, and global entities to ensure reliable supply.- Simplification of procurement processes for investigational drugs.- Legislative action to address shortages. | Delayed or interrupted treatments.- Increased risk of treatment failure and resistance development. Use of suboptimal regimens leading to adverse effects. | Increased drug costs due to scarcity and procurement challenges.- Higher healthcare system burden from prolonged treatments and complex regimens.- Inefficiencies in resource allocation and staff time.- Potential economic losses from increased TB transmission in communities. |  |
| Ballif et al 2014; Global^70^ | Detection and management of drug-resistant tuberculosis in HIV-infected patients from lower income countries | Cross-sectional study using questionnaires for program-level data and patient-level data from ART programs in lower-income countries. | To describe practices in the prevention and management of drug-resistant TB in HIV-infected individuals under ART programs in lower-income countries. | HIV-infected adults with tuberculosis (TB), receiving ART in lower-income countries (Southern Africa, East Africa, West Africa, Central Africa, Latin America, Asia-Pacific). | 553/449 | Program-level data from 47 ART programs; Patient-level data from 1,002 adult TB patients at 40 participating ART programs. | Second-line anti-TB drugs (specific drugs not detailed in the summary). | Lack of communications, Low stocks or expired drugs | Not mentioned | Not Mentioned | Reported drug shortages in 38% of ART programs; lack of access to second-line anti-TB regimens in 30% of ART programs. | Not mentioned |  |
| Singh et al 2017; India^38^ | Isoniazid Preventive Therapy among Children Living with Tuberculosis Patients: Is It Working? A Mixed-Method Study from Bhopal, India | Mixed-method study, including quantitative data collection (surveys) and qualitative insights (interviews). | To assess the uptake of IPT among children exposed to TB, evaluate barriers to its implementation, and suggest improvements for program effectiveness. | Children (under 5 years) in Bhopal, India, who are contacts of smear-positive TB patients. | 27/23 | 50 children, all living in households with at least one adult diagnosed with TB. | Isoniazid (100 mg Paediatric dose), the primary drug for preventive therapy. | Not mentioned | The stockouts were managed by prioritizing distribution to high-risk children and engaging community health workers for alternative solutions. | Not Mentioned | 6 Kids were not treated because of unavailability of INH. Inadequate IPT administration increases the risk of TB in children, undermining the goal of reducing childhood TB transmission and morbidity. | Not mentioned |  |
| Koomen et al 2019; South Africa^36^ | Effects and determinants of tuberculosis drug stockouts in South Africa | Cross-sectional study with regression analysis to assess stockout determinants and their impact on TB treatment outcomes across South Africa's districts. | To investigate determinants of TB drug stockouts and their effects on TB treatment outcomes such as cure, success, and death rates. | Patients undergoing TB treatment across 51 districts in South Africa. | Facility level data | Data aggregated at the district level from health facilities reporting TB drug stockouts; sample included 51 districts (out of 52). | TB drugs (specifics not listed but encompassing drugs necessary for treatment regimens). | Socioeconomic disparities (higher poverty correlated with higher stockouts), district income levels, and depot location (proximity to provincial depots increased stockout risk). | Not mentioned | Increase equity in socioeconomic factors, improve logistics to districts with higher poverty, and reform distribution systems from provincial depots. | Lower cure rates, lower success rates, and increased TB death rates in districts with higher stockout proportions. | Not mentioned |  |
| Sintayehu et al 2022; Ethiopia^28^ | Determinants ofÂ stock-outs ofÂ first line anti-tuberculosis drugs: theÂ case ofÂ public health facilities ofÂ Addis Ababa city administration health bureau, Addis Ababa, Ethiopia | A cross-sectional study using both qualitative and quantitative methods to assess the determinants of stockouts of first-line anti-TB drugs in Addis Ababa. | To identify and analyze the factors contributing to stockouts of first-line anti-TB drugs in public health facilities within Addis Ababa. | Public health facilities under Addis Ababa City Administration Health Bureau, serving patients in need of tuberculosis treatment. | Facility level data | 13 health facilities, with data from 67 health workers (including pharmacy managers and health officials). | Rifampicin, Isoniazid, Pyrazinamide, Ethambutol (first-line anti-TB drugs). | Poor forecasting and inventory management, delayed procurement, lack of communication between stakeholders, and funding gaps in the health sector. | Stockout management included emergency procurement, redistribution from other facilities, and coordination between health bureaus and stakeholders. | Strengthening forecasting and procurement processes, improving communication between health facilities and suppliers, and regular monitoring of stock levels to reduce stockouts. | Interruptions in TB treatment due to stockouts can lead to treatment failure, increased drug resistance, and higher mortality rates. | Stockouts lead to increased costs from emergency procurements and resource misallocation, impacting overall health system efficiency. |  |
| Otto-Knapp et al 2024; Europe^43^ | Availability of drugs for the treatment of multidrug resistant/rifampicin-resistant tuberculosis in the World Health Organization European Region, October 2023 | The report is observational and cross-sectional, based on data collected from WHO member countries in the European Region. | To assess the availability and accessibility of drugs for MDR-TB and RR-TB treatment, focusing on stockouts and shortages within the European Region. | The study covers countries in the WHO European Region. Populations affected include patients diagnosed with MDR-TB and RR-TB, particularly those in resource-constrained settings. | Not reported | The exact sample size is not specified, but the report includes data from several countries across the region, with a focus on the availability of essential drugs. | Bedaquiline, Delamanid, Linezolid, Clofazimine, and a few other second-line anti-TB drugs were reported to be in short supply or out of stock. | Issues include supply chain disruptions, funding limitations, regulatory delays, and the ongoing challenges of global health emergencies like COVID-19, which further strained drug distribution systems. | Countries reported using emergency procurement systems, adjusting treatment regimens where feasible, and prioritizing certain patient groups. Additionally, the use of alternative treatments was considered. | Strengthening procurement and supply chain management, increasing funding for MDR-TB treatment programs, expanding access to alternative medications, and enhancing global coordination. | Shortages significantly hindered the timely treatment of MDR-TB and RR-TB, leading to increased risks of treatment failure, resistance development, and further transmission of the disease. | The economic impact includes increased treatment costs due to the use of alternative drugs, higher hospitalization rates, and potential long-term health system burdens from unmanaged TB cases. |  |
| Nabity et al 2024; USA^35^ | Notes from the Field: Supply Interruptions of First- and Second-Line Oral Drugs to Treat Tuberculosis During the Previous 12 Months â€” California, Januaryâ€“March, 2023 | Retrospective survey | To investigate interruptions in the supply of first- and second-line oral drugs for tuberculosis (TB) in California, 2022. | Local TB programs in California, with varying TB incidences (average annual TB case counts ranged from 0 to 508). | Not mentioned | 61 local TB programs across California | First-line: Rifampin, Rifapentine, Rifabutin, Isoniazid, Pyrazinamide, Ethambutol; Second-line: Cycloserine, Ethionamide, Levofloxacin, Linezolid, Moxifloxacin, Para-aminosalicylate, Pretomanid. | Manufacturing delays, disruptions in supply chains, and global procurement issues. | Adjustments in treatment regimens, use of alternative drugs, or treatment pauses. In some cases, centralized buffer supplies were utilized. | Strengthening drug supply chains, establishing buffer stocks, and improving forecasting for drug availability. | Delayed treatment initiation, changes in regimens, and preventable negative patient outcomes such as worsened health conditions. | Increased healthcare costs due to regimen changes, treatment delays, and programmatic adjustments. |  |
| Sreevidya et al 2025, India | Implementation of isoniazid preventive therapy among children  living with diagnosed pulmonary tuberculosis patients: A mixed  methods study from Mumbai, India | Mixed method study | Aims to contribute to the scientific community by addressing key  gaps in the implementation of IPT for children exposed to TB and identifying the associated challenges | Kids with diagnosed pulmonary tuberculosis patients | 37/48 | 96 kids | INH syrup children | Not mentioned | Not mentioned | Not mentioned | Isoniazid preventive therapy discontinuation | Not mentioned |  |

**Table S2:** Summary of included evidence in the systematic review of drug stock‐outs in Human Immunodeficiency Virus (HIV), Tuberculosis, and Malaria Programs. Where sufficient empirical data were available, the reported impacts of stock-outs and any mitigation strategies to manage the shock-outs are presented. Where sufficient empirical data were not provided due to the type of evidence included, we have provided a summary of key relevant findings.

| **Causes of Shortages (n)** | **Region (n)** | **Countries (n)** | **Income Status** |
| --- | --- | --- | --- |
| **Anti-retroviral drug stockout** | | | |
| Supply Chain Issues (12) | Africa (10) | Cameroon (2)  Ghana (1)  Côte d'Ivoire (1)  South Africa (1)  Uganda (2)  Tanzania (1)  Kenya (1) | Lower middle-income country (LMIC)  LMIC  LMIC  Upper middle-income country (UMIC)  LMIC  LMIC  LMIC |
|  | Latin America (1) | Latin America (1) |  |
|  | Europe (1) | Serbia & Montenegro (1) | UMIC |
| Procurement Issues (9) | Africa (8) | Ghana (1)  Cameroon (1)  Côte d'Ivoire (1)  South Africa (1)  Nigeria (1)  Uganda (2)  Tanzania (1) | LMIC  LMIC  LMIC  UMIC  LMIC  LMIC  LMIC |
|  | Latin America (1) | Latin America (1) |  |
| Funding Issues (7) | Africa (7) | Ghana (1)  Uganda (4)  Côte d'Ivoire (1)  Central African Republic (1) | LMIC  LMIC  LMIC |
| Governance (3) | Africa (2) | Côte d'Ivoire (1)  Ghana (1) | LMIC  LMIC |
|  | Latin America (1) | Venezuela (1) | LMIC |
| Manufacturing Issues (2) | Latin America (1) |  |  |
|  | Australia (1) |  | High income country (HIC) |
| Weak Healthcare infrastructure (2) | Africa (2) | Nigeria (1)  Cameroon (1) | LMIC  LMIC |
| Forecasting and Inventory Management (3) | Africa (3) | Tanzania (1)  Uganda (2) | LMIC  LMIC |
| **Anti-Malarial drug stockout** | | | |
| Supply Chain Issues (4) | Africa (3) | Ethiopia (1)  Tanzania (1)  Kenya (1) | LMIC  LMIC  LMIC |
|  | North America (1) | United States of America (USA) (1) | HIC |
|  |  |  |  |
| Procurement Issues (3) | Africa (3) | Kenya (2)  Malawi (1) | LMIC  Lower income country (LIC) |
| Funding Issues (2) | Africa (2) | Kenya (2) | LMIC |
| Governance (3) | Africa (1) | Tanzania (1) | LMIC |
|  | North America (1) | USA (1) | HIC |
|  | Asia (1) | Pakistan (1) | LMIC |
| Forecasting and Inventory Management (5) | Africa (4) | Ethiopia (1)  Kenya (1)  Malawi (2) | LMIC  LMIC  LIC |
|  | Asia (1) | Pakistan (1) | LMIC |
| **Anti-Tubercular drug stockout** | | | |
| Supply Chain Issues (3) | Europe (1) | Europe (1) | HIC |
|  | North America (1) | USA (1) | HIC |
|  | United Kingdom (1) | United Kingdom (1) (1) | HIC |
|  |  |  |  |
| Procurement Issues (3) | Africa (1) | Ethiopia (1) | LMIC |
|  | North America (1) | USA (1) | HIC |
|  | United Kingdom (1) | United Kingdom (1) | HIC |
| Funding Issues (2) | Africa (1) | Ethiopia (1) | LMIC |
|  | Europe (1) | Europe (1) | HIC |
| Governance (3) | Africa (1) | Ethiopia (1) | LMIC |
|  | Europe (1) | Europe (1) | HIC |
|  | United Kingdom (1) | United Kingdom (1) | HIC |
|  |  |  |  |

**Table S3:** Distribution of studies reporting stock-outs by disease category, geographic region, country, and income classification
